# Computational drug repurposing of Akt-1 allosteric inhibitors for non-small cell lung cancer

**DOI:** 10.1038/s41598-023-35122-7

**Published:** 2023-05-16

**Authors:** Krishnaprasad Baby, Swastika Maity, Chetan Hasmukh Mehta, Usha Y. Nayak, Gautham G. Shenoy, Karkala Sreedhara Ranganath Pai, Kuzhuvelil B. Harikumar, Yogendra Nayak

**Affiliations:** 1 Department of Pharmacology, Manipal College of Pharmaceutical Sciences, Manipal Academy of Higher Education, Manipal, Karnataka 576104 India; 2 Department of Pharmaceutics, Manipal College of Pharmaceutical Sciences, Manipal Academy of Higher Education, Manipal, Karnataka 576104 India; 3 Department of Pharmaceutical Chemistry, Manipal College of Pharmaceutical Sciences, Manipal Academy of Higher Education, Manipal, Karnataka 576 104 India; 4 Cancer Research Program, Rajiv Gandhi Centre for Biotechnology (RGCB), Thiruvananthapuram, Kerala 695014 India

**Keywords:** Cancer, Chemical biology, Computational biology and bioinformatics, Drug discovery

## Abstract

Non-small cell lung carcinomas (NSCLC) are the predominant form of lung malignancy and the reason for the highest number of cancer-related deaths. Widespread deregulation of Akt, a serine/threonine kinase, has been reported in NSCLC. Allosteric Akt inhibitors bind in the space separating the Pleckstrin homology (PH) and catalytic domains, typically with tryptophan residue (Trp-80). This could decrease the regulatory site phosphorylation by stabilizing the PH-in conformation. Hence, in this study, a computational investigation was undertaken to identify allosteric Akt-1 inhibitors from FDA-approved drugs. The molecules were docked at standard precision (SP) and extra-precision (XP), followed by Prime molecular mechanics—generalized Born surface area (MM-GBSA), and molecular dynamics (MD) simulations on selected hits. Post XP-docking, fourteen best hits were identified from a library of 2115 optimized FDA-approved compounds, demonstrating several beneficial interactions such as pi–pi stacking, pi-cation, direct, and water-bridged hydrogen bonds with the crucial residues (Trp-80 and Tyr-272) and several amino acid residues in the allosteric ligand-binding pocket of Akt-1. Subsequent MD simulations to verify the stability of chosen drugs to the Akt-1 allosteric site showed valganciclovir, dasatinib, indacaterol, and novobiocin to have high stability. Further, predictions for possible biological interactions were performed using computational tools such as ProTox-II, CLC-Pred, and PASSOnline. The shortlisted drugs open a new class of allosteric Akt-1 inhibitors for the therapy of NSCLC.

## Introduction

Primary bronchogenic carcinoma, widely recognized as lung cancer, is a deadly tumour originating from the respiratory mucosa or respiratory glands^1^. According to the latest GLOBOCAN estimates, globally, lung cancer is the leading cancer cause in terms of incidence and mortality, with roughly 1.8 million new deaths (11.4%) and 2.2 million new diagnoses (18%), representing a severe health hazard. The number of new cases and deaths tends to rise every year^2^. Lung malignancies are predominantly non-small cell lung cancer (NSCLC), with nearly 85% of patients falling into this subtype, scategorized as adenocarcinoma (LUAD), squamous cell carcinoma (LUSC), and large cell carcinoma^3^. Despite advances, there was no significant reduction in incidence and mortality rates. Cancer cells spreads and metastasize relatively faster in NSCLC; most diagnoses happen near the late stages. The average 5-year survival rate in NSCLC is ~ 15%, the lowest of all cancer types, and hence there is a need for finding novel robust therapies for the disease^4^.

The serine/threonine kinase Akt, better known as protein kinase B, forms the phosphoinositide 3-kinase (PI3K) effector in the PI3K-Akt-mTOR signaling cascade^5^. This pathway intersects multiple signaling, and Akt forms a vital intermediary in cell survival mediated through growth factors^6^. Several oncologic proteins and tumour suppressor genes interconnect with the Akt pathway and contribute to cell size/growth, proliferation, and survival^7^. Three Akt isoforms are recognised, namely Akt-1/PKBα, Akt-2/PKBβ, and Akt-3/PKBγ, having a similar structure^8^. Dysregulation of this kinase plays a pivotal role in the onset and progression of several human cancers^9^. In NSCLC, the Akt-1 subtype plays a critical role in cell growth and migration and has been recognized widely as an anti-cancer target^10^. Akt isoforms have an N-terminal Pleckstrin homology (PH) domain, which is critical for membrane recruitment, attached to a central catalytic kinase domain (KD), parted by a hinge region (linker), which is 39-amino-acid long and a carboxyl-terminal (C-lobe) regulatory domain that harbours the hydrophobic motif (HM). The KD and C-terminal regulatory regions are essential for kinase activation. Unstimulated Akt appears auto-inhibited in the cytoplasm of cells, defined by the intramolecular interaction between its PH domain and the substrate-binding cleft of its kinase domain. Akt activity appears triggered via the association of its PH domain through a lipid or secondary messenger called the phosphatidylinositol 3,4,5-trisphosphate (PIP3), subsequently phosphorylation of threonine 308 (Thr-308) in its catalytic loop through phosphoinositide-dependent kinase 1 (PDK1), and serine 473 (Ser-473) phosphorylation in the HM separately by the mTORC2 complex. The Ser-473 phosphorylation encourages and enables an HM transition, stabilizing the phosphatase-resistant active conformation with catalytic activity^11^.

Repurposing drugs have been proven helpful in identifying potential chemotherapeutics for chronic use and have developed as an exciting strategy. These also offer considerable benefits by saving money, time, and resources^12^. The application of computational techniques has considerably reduced costs as there are fewer restrictions, in the way, in predicting potential binding sites in terms of complementarity between ligand and the protein target^13^. The allosteric inhibitors of Akt bind at sites other than active-site, positioned at the PH and kinase interface domain, roughly 10 Å away from the adenine pocket. In this mode, the Akt PH domain occupies space partly filled by the αC helix and the activation loop, which prevents the side of the kinase core from containing the activation loop. This way, the PH-domain masks the active site, sstabilizing the kinase in an inactive (PH-in) conformation. As a result, the PIP3-mediated recruitment of the Akt to the cell membrane blocks the phosphorylation of Thr308 and, eventually, kinase stimulation^14^. Moreover, this binding mode does not activate the feedback activation seen with the ATP competitive inhibitors^15^. These agents are advantageous over ATP-competitive inhibitors because they present improved specificity, lowered adverse reactions, and lesser toxicity^16^. MK-2206 was the initial selective Akt allosteric inhibitor in clinical trials. This molecule targeted the inactive kinase conformation and demonstrated encouraging proof of Akt signaling inhibition and tolerability in its first clinical experimentation^17^. Hence, this study aims to identify potential allosteric inhibitors of Akt-1 from a library of FDA-approved drugs using molecular docking and molecular dynamics (MD) simulations.

## Materials and methods

### Software and hardware specifications for computational simulations

The Schrödinger Small-Molecule Drug Discovery Suite was used for performing this computational study. The computational simulation experiments were performed in Maestro, the unified graphical interface of Schrödinger. Computational methods were as per earlier reports with little modifications^18–21^. In this computational study, various modules were used in the Maestro, including Protein Preparation Wizard (PPW), Prime, Glide and Desmond.

### Selection of target protein

The target protein considered for this computational study was Akt-1, which was in an autoinhibited form, co-scrystallized with an allosteric inhibitor-12j/0R4. The crystal structures of Akt-1 in the auto-inhibited form were searched in the Protein Data Bank (PDB) (http://www.rcsb.org/)^22^. In the RCSB database, we could find three PDB IDs, 3O96, 4EJN and 5KCV, as protein structures wherein Akt-1 was co-scrystallized with different allosteric inhibitors. Among them, PDB ID: 4EJN, co-crystallized with a ligand 12j/0R4, was selected for this computational study. The same crystal structure was being used for another computational study that involved the identification of imidazopyridine analogues as Akt-1 allosteric inhibitors by molecular docking and MD simulations by Gu et al.^23^.

### Protein preparation

The PDB-ID 4EJN of Akt-1 bound to co-crystal ligand (12j/0R4, IC_50_ = 5 nM) with a resolution of 2.19 Å was downloaded and imported to the Maestro interface^24^. The procedure followed by Gu et al., 2021 for screening and establishment of imidazopyridine analogues Akt1 allosteric inhibitors using 3D-QSAR, molecular docking and molecular dynamics simulations^23^ was followed by us for protein preparations. An initial setup of protein preparation was performed using the protein preparation wizard (PPW) as the downloaded protein cannot be used directly for computational calculations^25^. In the initial step, pre-processing of the protein structure was done by assigning bond orders, adding hydrogen, creating disulfide bonds, and filling the missing side chains and loops with the Prime tool. The termini were capped and water molecules beyond 5 Å were removed from the hetero group to rule out the hindrance. The different hetero atoms and unwanted crystalographic water molecules were removed from the crystal structure to refine the chain. The protonation states of ligands and residues was set using the PROPKA tool so that it helps  to simulate the exact experimental conditions. Under the OPLS3e force field, the refined protonated structure minimization generated a lower energy protein^26,27^.

### Ligand preparation

The DrugBank database (http://www.drugbank.ca) was used to identify 2115 US FDA-approved drugs which form the ligands in the docking study. The ligand structures were optimized using the LigPrep tool^28^. The ligands were prepared at pH 7.0 ± 2.0 by the Epik module, retaining the specific chiralities, desalting and generating the tautomers. Ultimately, the 2D structures were converted to 3D, which were geometry minimized under the OPLS3e forcefield for producing the lowest energy state 3D conformation of compounds with all the required corrections^29^. The optimized ligands were used in the docking calculations.

### Search for the binding site and molecular docking

The receptor grid generation tool specified the docking site for ligands in the protein structure in the Glide module surrounding the co-crystal ligand. In generating the receptor grid, the co-scrystallized ligand was picked from the minimized protein structure to eliminate it so that it would not be a part of the ligand-receptor docking. The receptor grid was generated using the default values of Vander Waal's radius scaling factor of 1 Å with a partial charge cut-off of 0.25 Å. Flexible docking was conducted using the Glide module, wherein the prepared ligands were to fit into the receptor grid at Standard Precision (SP) docking. The output of SP docking was put forward in extra precision (XP) to save the single best pose for each molecule^30^. Flexible docking algorithms generated the docking poses with docking scores and interaction with catalytic and other active site residues^31^. The shortlisted ligands that showed a better affinity towards the Akt-1 allosteric site were identified based on the molecular interactions with the ligands, the target protein, and the docking score.

### Validation of the docked poses

An extra precision (XP) Glide docking approach was used to assess the correctness of docking process. The co-crystallized ligand as well as other previously reported allosteric Akt-1 inhibitors were redocked to the binding site was docked with Glide XP docking mode. The bonding contacts identified after redocking and also the associations documented in the literature were utilised to confirm the docking results^32^.

### Free ligand binding energy calculations

Prime Molecular Mechanics/Generalized Born Surface Area (MM-GBSA) method was used to calculate the binding free energy of the protein–ligand complexes as an indication of the stability of the ligand-target complex. This attempt refined and rescored the docking results by incorporating molecular mechanics (MM) force fields combined with a generalized Born and surface area continuum (implicit) solvation model. The free binding energy calculation of the shortlisted molecules/hits was executed by incorporating a variable dielectric VSGB 2.0 solvation model,OPLS3e force field and the energy svisualizer in the prime module^33,34^.

### MD simulation

The eight best ligand–protein complexes shortlisted were subjected to MD simulation using Desmond module^35^. The XP-docked ligand–protein complex was subjected to an MD simulation for 100 ns with periodic boundary conditions in the Desmond module. The system builder tool created an orthorhombic box to submerge the protein–ligand complex in TIP3P explicit water model. Every atom in the ligand–protein complex was located at a minimum distance of 10 Å fixed between the complex and the box wall. A fair number of counter ions (sodium and chloride) been added to neutralize the system, and physiological or isosmotic conditions were emulated by adding 150 mM NaCl to the simulation box. The system energy was sminimized to align the protein structure within the simulation boundaries comfortably. The whole system was minimized with 2000 iterations with convergence criteria of 1 kcal/mol/Å using an OPLS3e force field. A predefined equilibration protocol was run before the production run of the simulation. MD simulation was performed under NPT conditions at a temperature of 300 K and 1.013 bar pressure. The temperature and pressure were maintained using a Nose–Hoover-Chain thermostat and Martyna–Tobias–Klein barostat. The energy and structure were recorded and saved in the trajectory every 10 ps, with 1000 frames saved to the trajectory.

After the MD simulation, the stability of the ligand–protein complex was determined from the Simulation Interaction Diagram (SID) generated from the simulation trajectory. Other parameters described in the SID like the RMSD, measured as an average change in the displacement of selected atoms of a defined frame to the reference frame and several other parameters were calculated.

### Prediction of toxicity, cell line cytotoxicity and biological activity prediction

The SMILES representation of compounds abstracted from the PubChem database (https://pubchem.ncbi.nlm.nih.gov/) submitted to the Prediction of Toxicity of Chemicals (ProTox-II). This web-based tool relies on molecular similarity, pharmacophores, fragment propensities, and machine-learning predictive models. A freely accessible toxicity estimation software tool (TEST) was used to predict small molecules relative in silico toxicity. Various toxicological endpoints, including hepatoxicity, carcinogenicity, immunotoxicity, mutagenicity, and cytotoxicity, were predicted from this tool^36^. A naive Bayes approach to predict cytotoxicity against the tumour and non-tumour cell lines was made from Cell-Line Cytotoxicity Predictor (CLC-Pred) (http://www.way2drug.com/Cell-line/) webserver at the pharmacological activity (Pa) and pharmacological inactivity (Pi), with the criteria Pa > Pi (suggested to be active)^37^. Prediction of Activity Spectra for Substances Online (PASSOnline), (http://www.way2drug.com/PASSOnline), a web-based server, uses Bayesian analysis to predict biological activity, including pharmacological effects, mechanisms of action based on the structural formula of the chemical^38^.

### Ethics declaration

The work does not include human, animal or biologicals.

## Results and discussion

### Protein structure

PI3K-Akt-mTOR signalling is activated aberrantly in many cancers, including lung cancers. Akt is expressed frequently in lung cancer, resulting in an abnormal increase in phosphorylated level. This has contributed to tumour progression through increased cell growth, migration, invasion, and drug resistance development by the phosphorylation of its downstream substrates, which makes Akt an appealing cancer target^39^. An inhibition of Akt activity in cells induced cell death. Allosteric or ATP-competitive inhibitors are the ways to achieve Akt inhibition, and many molecules are in clinical development. Recent research shows that allosteric Akt inhibitors engage at the PH and kinase domain interface with remarkable pharmacological selectivity, limiting non-catalytic Akt activity like acetate excretion^40^.

The crystal structure coordinate 4EJN, downloaded from the PDB, was used for this computational study. The selection was based solely on parameters like resolution (2.19 Å), source organism (*homo sapiens*), receptor comprehensiveness, expression system (*Spodoptera frugiperda*), R-value free (0.276), R-factor (0.237), and had a co-crystal ligand (12j/ 0R4, IC_50_ = 5 nM). The chain A of crystal structure 4EJN had a sequence length of 446 amino acids. The co-crystallized ligand was identified near the junction between PH and kinase domains, about 10 Å away ATP binding pocket, making several polar and non-polar contacts. The core of 12j made a hydrophobic contact with conserved tryptophan (Trp-80) residue. The ring-nitrogen interacted via a water-mediated hydrogen bond network to the aspartic acid residue of the DFG- motif (Asp-292). The phenyl ring of the ligand interacted directly hydrophobically with the conserved Tyr-272 residue. A hydrophobic interaction was observed with the aromatic core and the Val-270 residue. The contact between the PH and Akt-1 kinase domains rendered the kinase catalytically inactive and closed. The lipid binding site appeared occluded by the catalytic domain. As a result, we assumed that Akt-1 would make an appealing drug target, and we used docking and MD simulations to find allosteric Akt-1 inhibitors.

### Ligand docking

Computational studies such as molecular or ligand docking had been performed to identify potent hits against Akt-1. Using computational technologies, in silico drug identification reduces millions of chemicals to a few possible ones against a target quickly and effectively^41^. Computational studies allow the prediction or study of the intermolecular interactions as the significant factors that could significantly impact the affinity of a ligand for a receptor. Hence, a chemical library of 2115 FDA-approved molecules, whose structures were soptimized using LigPrep, was used for this study. After identifying the binding site was defined at default dimensions around the co-crystal ligand on the linkage of the PH domain and the kinase domain interface employing the receptor grid generation tool.

After introducing the optimized ligands to the virtual space, SP-docking was performed to shortlist top hits. Based on their docking score, the top hits underwent a flexible docking approach at XP to rank the ligand–protein complexes. Glide SP could determine the binding ability by adding the interaction energies from several compounds contributing to the scoring matrix^30,31^. With Glide, XP scoring seems to be the most precise since it incorporates more extensive assessment and filtering than SP. It either rewards or penalizes hydrogen bonds, hydrophobic contacts, and π-cations. The docking score could provide only a statistically valid and semiquantitative rating of a ligand's ability to bind to a specific receptor geometry. It also help in removing false-positive results via a statistically valid and semiquantitative rating^42^.

Docking the molecules using SP and XP revealed both binding affinity and orientations of ligand–protein associations in space that could impede a protein function. Some molecules were shortlisted to perform further studies. All the shortlisted hits showed a significant interaction with the conserved residues Trp-80, or Tyr-272 and other critical amino acid residues in the kinase hinge region that could contribute to the allosteric inhibition. The molecules with the lowest glide score value were considered the best docked and potent lead molecules. The XP-docking score of the shortlisted ligand–protein complex varied from −12.121 to −6.911 (Table 1). Table 2 shows the docking score, glide score, Glide Van der Waal (G_evdw_), Glide energy (G_energy_), Glide model (G_model_), Glide ECoulomb (G_ecoulomb_) and Glide eInternal (G_einternal_) of the top-14 ligands, namely vilazodone, indacaterol, pitavastatin, nomegestrol, raltitrexed, novobiocin, ezetimibe, ditazole, nebivolol floxuridine, delorazepam, valganciclovir, dasatinib, and lorazepam.

**Table 1:** Docking score and intermolecular interactions with amino acid residues at Akt-1 allosteric pocket by top-14 drugs.

Name of molecule	Docking score	2D representation of molecular interactions
1. Vilazodone	−12.121	π–π stacking: Trp-80 H-bonds: Asn-54, Thr-211 Salt bridge: Asp-292 Polar: Gln-79, Thr-82, Gln-203, Asn-204, Ser-205 Charged (−)ve: Glu-17, Asp-274 Charged (+)ve: Arg-86, Lys-268, Arg-273 Hydrophobic: Phe-55, Ile-84, Leu-210, Ala-212, Leu-264, Val-270, Val-271, Tyr-272, Ile-290
2. Indacaterol	−10.413	π–π stacking: Trp-80 H-bonds: Thr-211 Salt bridge: Asp-292 Polar: Asn-54, Gln-79, Thr-81, Thr-82, Gln-203, Asn-204, Ser-205, Asn-279 Charged (−)ve: Asp-274 Charged (+)ve: Asp-274, Lys-268 Hydrophobic: Ile-84, Leu-210, Ala-212, Leu-213, Leu-264,Val-270, Val-271, Tyr-272, Ile-290
3. Pitavastatin	−10.375	π–π stacking: Trp-80 H-bonds: Tyr-272, Asn-279, Phe-293 Polar: Gln-79, Ser-205, Thr-211, Thr-291 Charged (−)ve: Asp-274 Charged (+)ve: Lys-179, Lys-268, Arg-273 Hydrophobic: Ile84, Leu-210, Leu-264, Val-270, Val-271, Ile-290
4. Nomegestrol	−9.707	H-bond: Gln-79, Thr82, Ser-205 Charged (−)ve: Asp-292 Charged (+)ve: Lys-268 Polar: Asn-54, Thr-81, Gln-203, Asn-204, Thr-211 Hydrophobic: Trp-80, Leu-210, Ala-212, Leu-264, Val-270, Val-271, Tyr-272
5. Raltitrexed	−9.476	H-bond: Lys-276, Cys-296, Leu-295, Lys-307, Gly-311 Salt bridge: Lys-276, Lys-307 Hydrophobic: Phe-161, Phe-293, Cys-310 Charged (−)ve: Asp-274, Glu-278, Asp-292, Glu-298 Charged (+)ve: Lys-158, Arg-179, Arg-273, Lys-297 Polar: Thr-82, Asn-279, Thr-291, Thr-312
6. Novobiocin	−9.401	H-bond: Asp-292 Hydrophobic: Ile-84, Leu-210, Ala-212, Val-264, Val-270, Tyr-272, Ile-290, Tyr-326 Polar: Asn-54, Gln-79, Thr-81, Thr-82, Asn-204, Ser-205, Thr-211 Polar: Asn-54, Gln-79,Thr-81, Thr-82, Asn-54, Ser-205, Thr-211 Charged (−)ve: Asp-274 Charged (+)ve: Lys-268, Arg-273 Salt bridge: Asp-292
7. Ezetimibe	−9.184	π–π stacking: Trp-80 H-bond: Gln-79, Ile-290 Hydrophobic: Ile-84, Leu-210, Leu-264, Val-270, Val-271, Tyr-272, Phe-293 Polar: Asn-54, Thr-82, Asn-279, Gln-203, Ser-205, Asn-279, Thr-291 Charged (+)ve: Lys-268 Charged (−)ve: Asp-292, Asp-274 Glycine bond: Gly-294
8. Ditazole	−9.066	H-bond: Gln-79, Tyr-272 Hydrophobic: Trp-80, Ile-84, Leu-210, Leu-264, Val-270, Ile-290 Charged (+)ve: Lys-268, Arg-273 Charged (−)ve: Asp-274, Asp-292 Polar: Asn-54, Thr-82, Ser-205, Thr-211, Thr-291
9. Nebivolol	−8.920	π–π stacking: Trp-80 H-bond: Asp-292 Salt bridge: Asp-292 Hydrophobic: Ile-84, Leu-210, Ala-212, Leu-264, Val-270, Tyr-272, Ile-290, Tyr-326 Polar: Asn-54, Gln-79, Thr-81, Thr-82, Asn-204, Ser-205, Thr-211 Charged (−)ve: Asp-274 Charged (+)ve: Lys-268
10. Floxuridine	−8.888	π–π stacking: Trp-80 H-bond: Ser-205, Thr-211 Hydrophobic: Leu-210, Ala-212, Leu-213, Leu-264, Val-270, Ile-290, Ty-272 Charged (+)ve: Lys-268 Charged (−)ve: Asp-292
11. Delorazepam	−8.821	π–π stacking: Trp-80 H-bond: Thr-211 Charged (+)ve: Asp-292 Charged (−)ve: Lys-268 Polar: Asn-54, Gln-79, Thr-81, Thr-82, Asn-204, Ser-205 Hydrophobic: Leu-210, Ala-212, Leu-264, Val-270, Val-271, Tyr-272, Ile-290
12. Valganciclovir	−8.225	π–π stacking: Trp-80 H-bond: Thr-82, Ser-205, Thr-211, Tyr-272 Hydrophobic: Ile-84, Leu-210, Ala-212, Leu-213, Leu-264, Val-270, Val-271 Charged (−)ve: Asp-292 Charged (+) ve: Lys-268, Arg-273
13. Dasatinib	−7.409	π–π stacking: Trp-80 H-bonds: Asn-54, Tyr-272, Salt bridge: Asp-292 Polar: Gln-79, Thr-82, Gln-203, Asn-204, Ser-205, Thr-211 Charged (−)ve: Asp-274 Charged (+)ve: Lys-268, Arg-273 Hydrophobic: Tyr-18, Ile-84, Leu-210, Leu-264, Cys-310, Tyr-326 Halogen bond: Asn-54
14. Lorazepam	−6.911	H-bond: Ser-205 Hydrophobic: Trp-80, Leu210, Leu-264, Val-270, Tyr-272, Ile-290 Polar: Gln-79, Thr-81, Asn-204, Thr-211 Charged (−)ve: Asp-292 Charged (+)ve: Lys-268 Halogen bond: Thr-82

**Table 2:** Demonstrates the results of molecular docking as glide score, Glide Van der Waal (G_evdw_), Glide energy (G_energy_), Glide model (G_model_), Glide Ecoulomb (Ge_coulomb_) and Glide eInternal (G_einternal_) of the top-14 molecules.

Molecules	Glide g-score	Glide Van der Waal (G_evdw_)	Glide energy (G_energy_)	Glide model (G_model_)	Glide Ecoulomb (G_ecoulomb_)	Glide eInternalG (G_einternal_)
1. Vilazodone	−12.413	−51.664	−63.244	−98.862	−11.580	5.909
2. Indacaterol	−10.413	−46.103	−54.170	−87.771	−8.067	2.042
3. Pitavastatin	−10.375	−43.998	−54.161	−84.013	−10.162	6.125
4. Nomegestrol	−9.707	−35.321	−40.699	−51.489	−5.378	0.000
5. Raltitrexed	−9.476	−46.010	−65.201	−95.338	−19.191	12.578
6. Novobiocin	−9.423	−57.749	−64.908	−100.919	−7.159	11.899
7. Ezetimibe	−9.184	−48.452	−53.739	−81.660	−5.287	4.810
8. Ditazole	−9.066	−36.513	−45.979	−62.939	−9.466	10.813
9. Nebivolol	−8.985	−39.702	−48.684	−78.428	−8.982	3.378
10. Floxuridine	−8.991	−26.490	−31.871	−44.070	−5.381	1.907
11. Delorazepam	−8.821	−37.835	−41.497	−57.292	−3.662	0.182
12. Valganciclovir	−8.530	−42.727	−51.724	−74.501	−8.998	6.338
13. Dasatinib	−7.409	−49.923	−53.975	−84.827	−4.052	8.565
14. Lorazepam	−6.911	−39.392	−42.142	−56.807	−2.750	3.693

**Figure 1:**
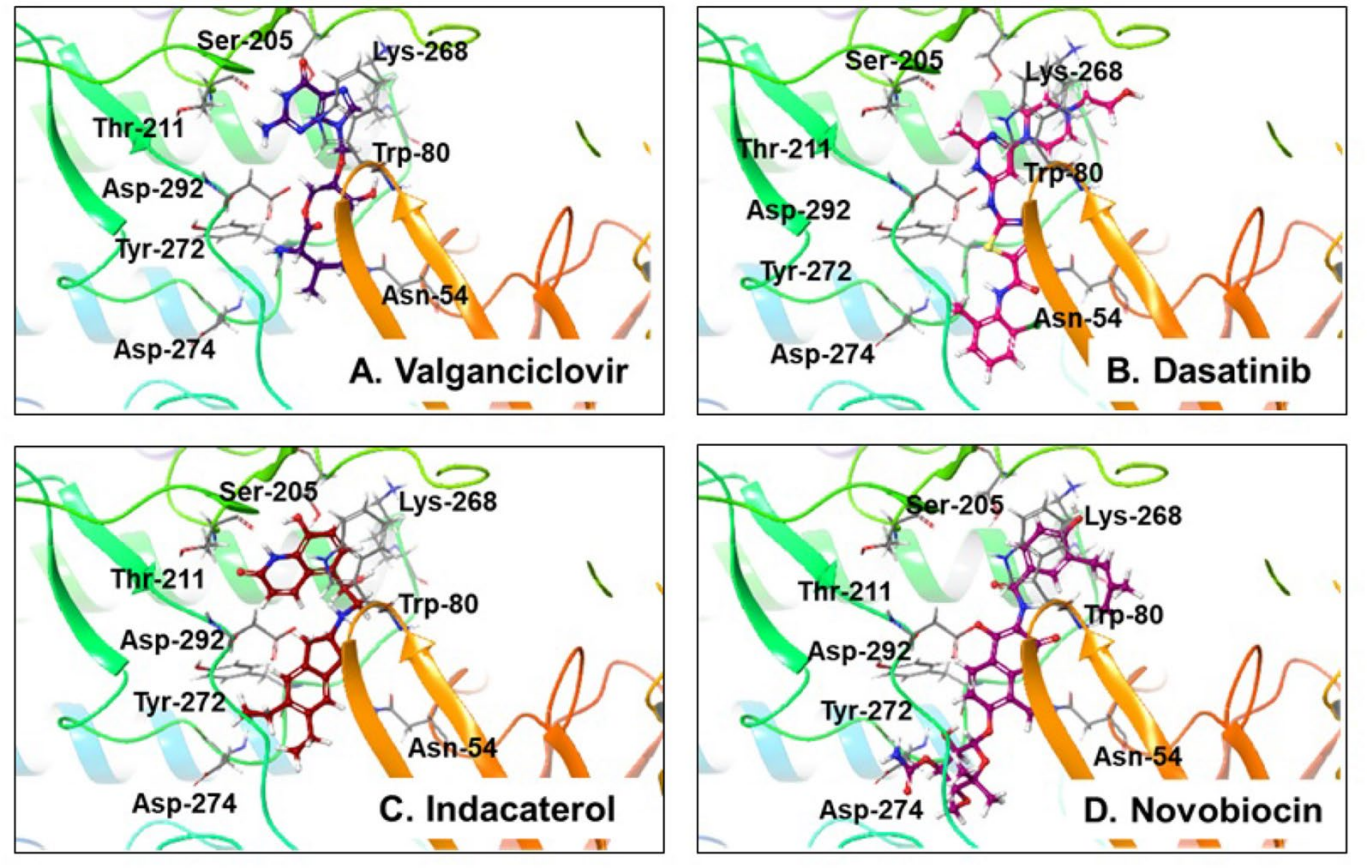
Various ligand–protein interactions displayed by top-4 molecules with the Akt-1 allosteric site (PDB ID: 4EJN). (The remaining ten molecule interactions are represented in the supplementary-1 material, Fig. 1S).

Upon visual observations of molecular interactions and MD simulations, the 3D structures of top-4 docked ligand–protein complexes and their molecular interactions with amino acids are represented in Fig. 1. The remaining details of ten molecules of MD simulations are included in the supplementary-1 materials (Fig. 1S).

Allosteric inhibitors interact with and engage outside ATP binding pocket, resulting in a closed activation loop conformation. Studies have demonstrated that allosteric inhibitors of Akt bind and interact with the tryptophan residue (Trp-80) of the PH domain^14,43^. Computational docking analyses investigating the binding model by MK-2206 (an allosteric Ak1/2 inhibitor in clinical investigations), on Akt-1 revealed that the compound interacted with the residues Trp-80 and Tyr-272 along with other residues like Asn-53, Gln-59, Leu-78, Val-201, Leu-264, and Val-270 of Akt-1^44^. Generally, ligand–protein complexes sstabilize if more hydrophobic and polar contacts are exhibited in the docking. The similarity exhibited among interacting residues for the co-scrystallized and docked ligands, a high negative docking score, implies that they bind similarly and can interfere with and disrupt the target protein function in the same way as observed with the co-scrystallized ligand. Thus, the docked complexes could favour the PH-in conformation and inactive or autoinhibited state of Akt-1, thereby preventing phosphorylation-mediated kinase activation. Our findings, therefore, provide evidence that identified compounds might suppress Akt-1 by functioning as an allosteric inhibitor by maintaining the favoured closed conformation necessary for its activity.

## Validation of molecular docking

The docking protocol employed was docking the co-crystallized ligand, and other reported allosteric Akt-1/2 inhibitors (MK-2206, ARQ-02 (Miransertib), TAS-117 and BAY 1125976) to the earlier generated receptor grid. The binding mode of the above mentioned ligands is reported in supplementary-2 file. The molecules were docked at XP-mode using the Glide module to the generated receptor grid. Interpretation of the results demonstrated interactions between amino acid residues. The interactions with Trp-80, Val-270, Val-271, Tyr-272 and Asp-292 appear common among the docked complexes.

### Free ligand binding energy calculations

The docking studies experiments generated false-positive findings for binding affinities among a protein and a ligand, which might explain why the ligand and the protein complexes. Poisson-Boltzmann surface area (MM-GBSA), binding free energy estimates of different ligand–protein complexes to represent the associations for distinct protein and ligand are being used to represent binding free energies (ΔG) as molecular mechanics estimates^45^. From the results of Glide docking and investigation of their 2D and 3D binding mode, interactions made with residues in the allosteric site, 14 docked ligand–protein complexes had been recognized to have a better or good affinity with the Akt-1 allosteric site. These docked ligand–protein complexes were shortlisted for further study. To assess the relative potency against the receptor, top-14 ligand–protein complex from XP-docking were taken forward for free ligand energy calculation (ΔG), using Prime MM-GBSA. These energy values help us understand the energy contributions of the stability factor of the individual ligand–protein complexes. Among the ligand–protein complexes, dasatinib possessed better inhibitory activity against the chosen receptor (ΔGbind −69.74 kcal/mol). Other energy components for the dasatinib-Akt-1 complex include Coulombs energy (ΔG_cou_ 53.96 kcal/mol), van der Waals (ΔG_vdW_ −61.13 kcal/mol), and solvation energy (ΔG_solv_ −42.12 kcal/mol), Covalent energy (ΔG_cov_ 7.31 kcal/mol) and lipophilic energy (ΔG_lipo_ −21.65 kcal/mol). The binding energy contributions of many other ligand–protein complexes are represented in Table 3.

**Table 3:** Binding free energy (Prime MM-GBSA) change profiles of the chosen top-14 molecules with Akt-1.

Molecules	Δ dG bind	ΔG Bind coulomb	ΔG Bind covalent	ΔG Bind lipo	ΔG Bind solv GB	ΔG Bind vdW
	(Kcal/mol)					
1. Vilazodone	−57.00	9.29	9.09	−19.93	11.76	−58.94
2. Indacaterol	−56.11	17.65	6.25	−23.93	4.14	−53.30
3. Pitavastatin	−31.07	32.83	11.31	−29.55	11.40	−48.78
4. Nomegestrol	−48.63	−13.39	5.10	−25.77	31.64	−44.78
5. Raltitrexed	−32.07	34.35	7.22	−11.77	0.89	−57.39
6. Novobiocin	−36.02	−84.37	11.33	−26.91	140.12	−70.47
7. Ezetimibe	−49.56	−12.68	1.16	−26.76	47.99	−53.52
8. Ditazole	−55.16	−21.90	4.99	−25.49	32.23	−39.64
9. Nebivolol	−59.93	11.19	8.49	−28.02	6.43	−51.16
10. Floxuridine	−35.28	−18.80	4.99	−9.85	22.05	−28.09
11. Delorazepam	−50.34	−6.60	1.06	−22.78	21.18	−38.75
12. Valganciclovir	−42.16	−26.44	3.87	−12.15	50.82	−48.46
13. Dasatinib	−69.74	53.96	7.31	−21.65	−42.12	−61.13
14. Lorazepam	−49.13	−9.83	3.60	−22.76	21.34	−39.22

### MD simulation

**Figure 2:**
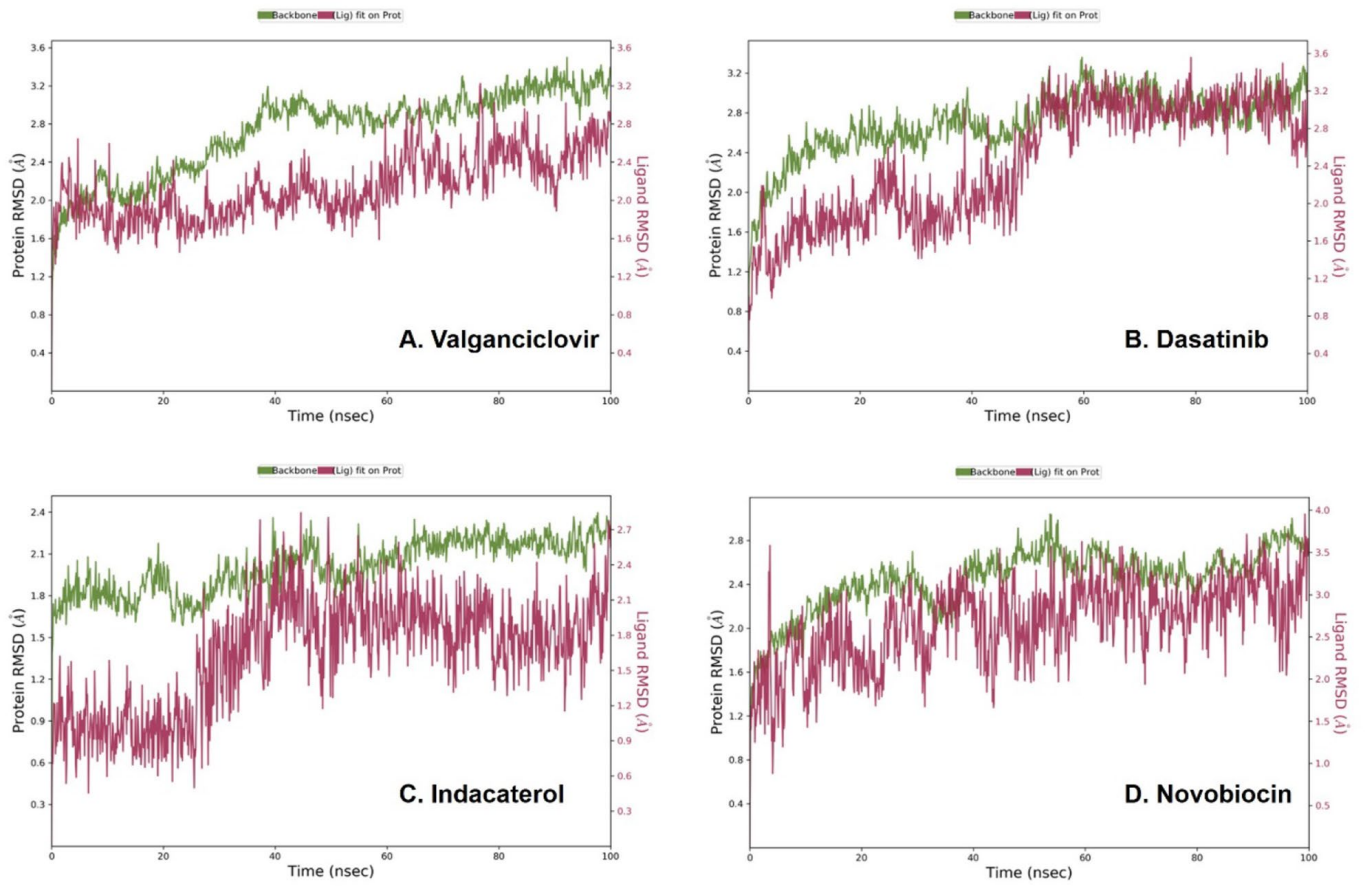
Results of Root Mean Square Deviation (RMSD) observed during MD simulation of shortlisted top-4 molecules for a specified frame relative to a reference frame. The RMSD plots for remaining 10 molecules are given along with the supplementary-1 material, Fig. 2S.

The SP and XP docking had many limitations, such as flexibility and complexity with the ligand flexibility, entropic effects, solvation/desolvation and influence of water molecules (and ions) during binding^46^. Hence, MD simulations were performed on the solvated system of the respective docked complexes using Desmond to sanalyze the binding stability as fluctuations in RMSD, followed by the persistence of protein–ligand interaction profiling during the 100 ns within the Akt-1 allosteric site mimicking biological conditions. Furthermore, MD simulations seek to simulate atom motions over some time. The RMSD for the protein is shown on the left y-axis, while the ligand RMSD profile matched on the protein structure backbone is given on the right x-axis. RMSD parameter is used to calculate the system's dynamical stability. The ligand, was bound in the binding pocket of a biomolecule, it is thought to impact the protein residue atoms by inducing flexibility. As a result, it represents a full measure of structural variations during MD simulation. The plot of the protein structure backbone counted throughout the MD simulation was matched the original structure to the enumeration of RMSD. A RMSD shift of 1–3 Å is acceptable during MD simulations for small protein complexes attached to ligands. However, a significantly high number implies a major conformational shift of the protein, and the system is not stable. The RMSD graphs from the MD simulations revealed considerable transformations for protein, ligand, and stability in contacts throughout the MD simulation, as seen in Fig. 2.

**Figure 3:**
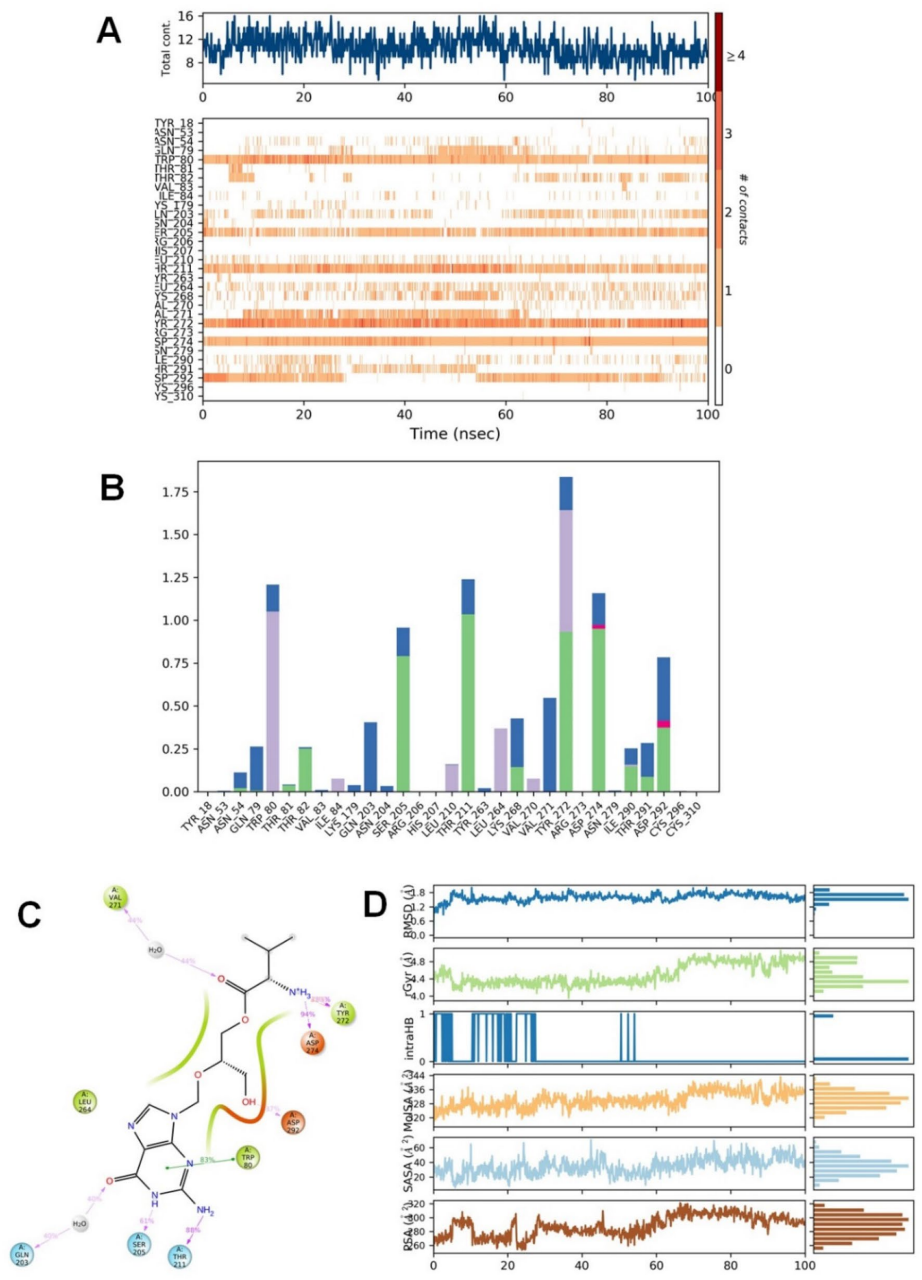
Protein interactions with the valganciclovir monitored during the course of trajectory (**A**) Timeline illustration of total specific contacts that several interacting residues makes with a ligand (**B**) Protein–ligand contacts described as histogram (**C**) Various protein- ligand interactions that stays for more than 30.0% of the simulation time (**D**) Various ligand properties in the form of ligand RMSD, radius of gyration (rGyr), molecular surface area (MolSA), solvent‐accessible surface area (SASA) and polar surface (PSA) expressed in Å during the 100 ns of the MD simulation.

Valganciclovir was bound to the allosteric site of Akt-1 and demonstrated both hydrophilic and hydrophobic contacts during MD simulation. After a preliminary fluctuation due to the equilibration for 35 ns, the protein structures RMSD varied between 0.7 and 3.4 Å until the simulation culminated (Fig. 2A). The protein structure oscillations between 2.7 Å indicated a stable protein structure where the complex had not suffered substantial changes in conformation. Similarly, the ligand RMSD varied from 1.6 to 2.9 Å up to the end of the simulation. The ligand structure fluctuations remained between 1.3 Å, demonstrating that the ligand is steadily bound to the kinase allosteric site and has not significantly diffused from the bound position. Figure 3 depicts the ligand–protein interactions and the protein–ligand contacts recorded throughout the MD simulation. Interactions that last more than 30% of the simulation time in the selected trajectory were considered. Various protein–ligand contacts such as H-bonds, Hydrophobic, Ionic, and Water bridges were observed during the simulation. The purine ring in the valganciclovir made π-π interactions with the conserved tryptophan (Trp-80) of Akt-1. This interaction persisted for more than 83% of the simulation time. The 6-oxo functionality demonstrated a water-bridged hydrogen bond with the Gln-203, which persisted for 40% of the simulation time. The N-1 of the purine ring demonstrated a direct hydrogen bonding interaction with Ser-205, which persisted for 61% of the simulation time. The 2-amino functional group in the purine ring made direct hydrogen bonding with the ligand, which persisted for 88% of the simulation time. The hydroxyl group in the 3-hydroxyl propyl chain demonstrated a direct hydrogen bonding interaction with the residue Asp-292, which was retained for 32% of the simulation time. The terminal 2-amino-3-methyl butanoate chain demonstrated both direct and water-bridged hydrogen bonding interactions. The carbonyl group in this chain demonstrated a water-bridged hydrogen bond with Val-271, which persisted for 44% of the simulation time. Similarly, the 2-amino functional group made two direct hydrogen bonding interactions, with Tyr-272 and Asp-274, which persisted for more than 90% and 94%, respectively. The Leu-264 residue made hydrophobic contacts, and among multiple residues, Tyr-272, Trp-80, Asp-274 and Thr-211 made frequent contact with the ligand. The interactions, Glu166, Gln189, and Thr26 of the docked pose, were retained during the 100 ns simulation time. The RMSD plot of valganciclovir shows peaks of around ~ 2.007 Å. The radius of gyration measured for the extendedness of the ligand, equivalent to its moment of inertia, shows peaks of around 5.052 Å. The MolSA was found between 316.156 and 343.261 Å. The SASA and PSA area ranged from 5.738 to 70.732 Å and 252.315 to 321.063 Å, respectively.

**Figure 4:**
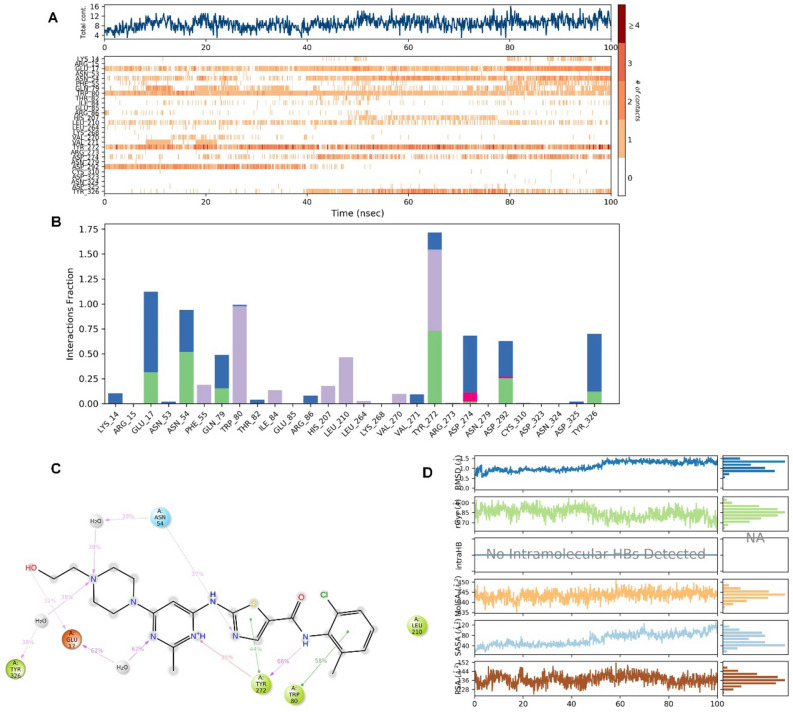
Protein interactions with the dasatinib monitored during the course of trajectory (**A**) Timeline illustration of total specific contacts that several interacting residues makes with a ligand (**B**) Protein-ligand contacts described as histogram (**C**) Various protein- ligand interactions that stays for more than 30.0% of the simulation time (**D**) Various ligand properties in the form of ligand RMSD, radius of gyration (rGyr), molecular surface area (MolSA), solvent‐accessible surface area (SASA) and polar surface (PSA) expressed in Å during the 100 ns of the MD simulation.

Dasatinib, a tyrosine kinase inhibitor for treating lymphoblastic or chronic myeloid leukaemia, was bound to the Akt-1 allosteric site, demonstrating several hydrophilic and hydrophobic interactions during the MD simulation. After the initial fluctuation due to the equilibration for 35 ns, the protein structures RMSD varied between 0.9 and 3.2 Å till the end of the simulation (Fig. 2B). The protein structure fluctuations between 2.3 Å indicated a stable protein structure where the complex had not undergone significant conformational changes. Similarly, the ligand structures RMSD varied between 2.0 and 3.2 Å till the end of the simulation. The ligand structure fluctuations remained between 1.2 Å, indicating that the ligand is stably bound to the kinase allosteric site and has not diffused significantly from the bound position. Figure 4 depicts the ligand–protein interactions and the protein–ligand contacts recorded throughout the MD simulation, wherein interactions that last more than 30% of the simulation time were considered. Several molecular interactions as H-bonds, hydrophobic, ionic, and water bridges, were observed as protein–ligand contacts during the simulation. The aromatic benzene ring with the 2-chloro-6-methyl and thiazole functional groups demonstrated the conserved residues Trp-80 and Tyr-272, respectively. Both of these interactions were proven te an essential for allosteric inhibition of molecules targeting the allosteric site of Akt-1. This interaction persisted for 58 and 44% of the simulation time. The hydroxy group in the 2-hydroxy ethyl tail demonstrated a direct hydrogen bonding interaction with residue Glu-17, which persisted for 31% of the simulation time. The N-3 of the 2-methyl-4-pyrimidyl part of the ligand demonstrated water-bridged hydrogen bonding interactions with Glu-17, which persisted for 62% of the simulation time. The N-4 of the piperazine group in the ligand demonstrated water-bridged hydrogen bonding interactions with the residues Asn-54 and Tyr-326, respectively, that persisted for 38% of the simulations. The N-1 of the 2-methyl-4-pyrimidyl portion of the ligand demonstrated a direct hydrogen bonding interaction with the Tyr-272 residue that persisted for 35% of the simulation time.

The heterocyclic nitrogen atom in the thiazole ring demonstrated a direct hydrogen bonding interaction with residue Asn-54, which persisted for 37% of the simulations. The nitrogen atom in the carboxamide linker connecting thiazole and 2-chloro-6-methyl phenyl groups demonstrated a direct hydrogen bonding interaction with Tyr-272 that persisted for 66% of the simulation time. The residues, Tyr-272 and Trp-80, had frequent contact with the ligand throughout the simulation. The RMSD plot of dasatinib shows peaks of around ~ 1.573 Å. The radius of gyration (rGyr) measured for the extendedness of the ligand, equivalent to its moment of inertia, shows peaks of around 6.07 Å. The MolSA was found to range from 435.266 to 450.709 Å. The SASA and PSA ranged from 11.78 to 132.367 and 123.734 to 151.451 Å, respectively.

**Figure 5:**
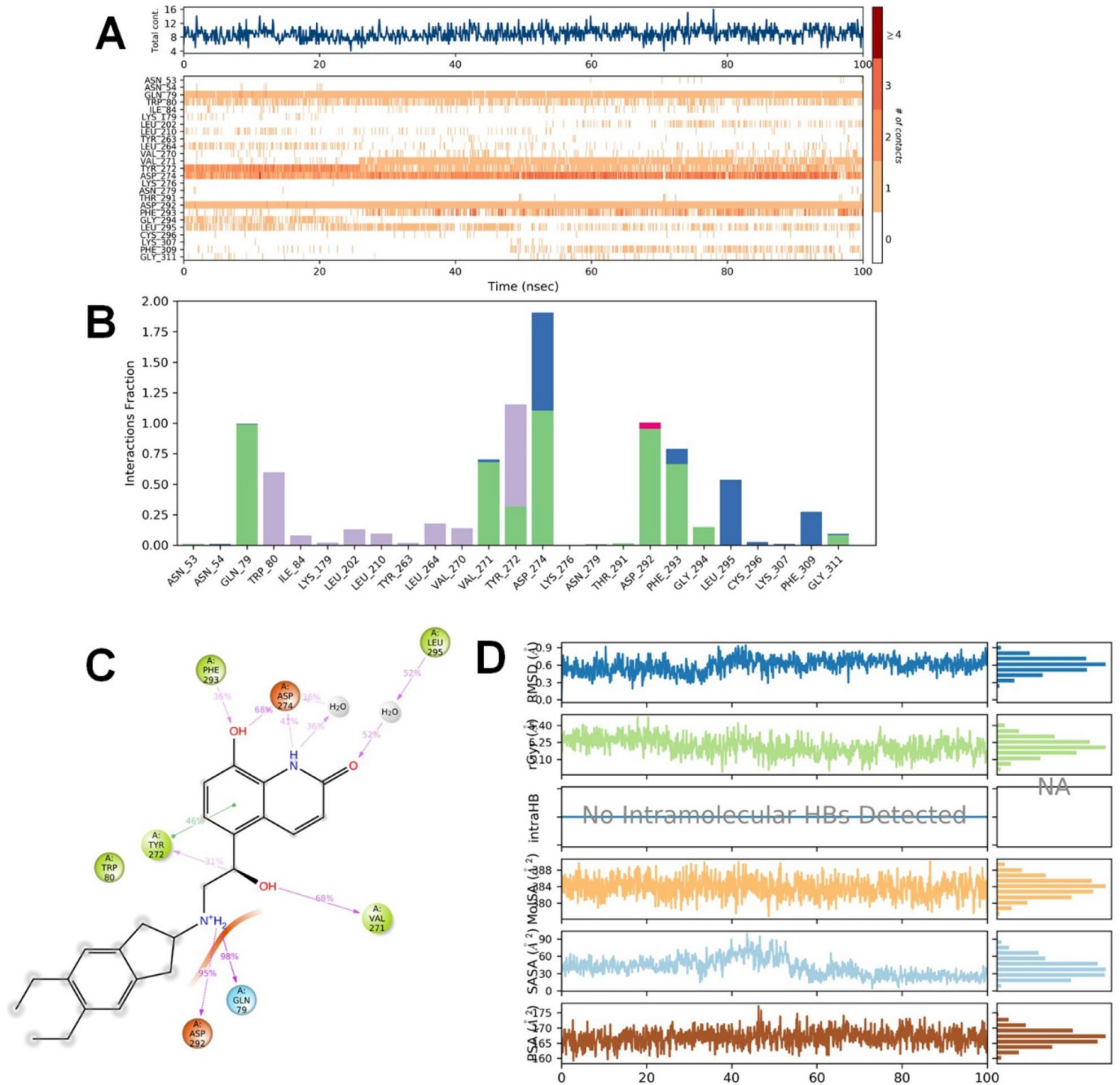
Protein interactions with the Indacaterol monitored during the course of trajectory (**A**) Timeline illustration of total specific contacts that several interacting residues makes with a ligand (**B**) Protein–ligand contacts described as histogram (**C**) Various protein- ligand interactions that stays for more than 30.0% of the simulation time (**D**) Various ligand properties in the form of ligand RMSD, radius of gyration (rGyr), molecular surface area (MolSA), solvent‐accessible surface area (SASA) and polar surface (PSA) expressed in Å during the 100 ns of the MD simulation.

Indacaterol is a β2 adrenergic agonist used to treat COPD and moderate to severe asthma. After the initial fluctuation due to the equilibration for 35 ns, the protein structures RMSD varied between 0.7 and 2.4 Å till the end of the simulation (Fig. 2C). The protein structure fluctuations between 1.7 Å indicated a stable protein structure where the complex had not undergone significant conformational changes. Similarly, the ligand structures RMSD varied between 1.8 and 2.7 Å till the end of the simulation. The ligand structure fluctuations remained between 0.9 Å, indicating that the ligand is stably bound to the kinase allosteric site and has not diffused significantly from the bound position. Figure 5 depicts the ligand–protein interactions and the protein–ligand contacts recorded throughout the MD simulation, wherein interactions that last more than 30% of the simulation time were considered. Several molecular interactions as H-bonds, hydrophobic, ionic, and water bridges, were observed as protein–ligand contacts during the simulation. The amino functional group near the hydroxyethyl linker demonstrated two direct hydrogen bonding interactions with residues, Gln-79 and Asp-292, which persisted for 98 and 95% of the simulation time. The hydroxyl functionality in the hydroxyethyl linker demonstrated direct hydrogen bonding interactions with the residue Val-271 and Tyr-272, which persisted for 68 and 31% of the simulation time. The benzene ring in the aromatic quinoline-2-one demonstrated a π-π interaction with the residue Tyr-272, which persisted for 46% of the simulation time. The 8-hydroxyl function group attached to the quinoline-2-one ring demonstrated direct hydrogen bonding interactions with Asp-274 and Phe-293, accounting for 68 and 36% of the simulation time. The N-1 of the quinoline-2-one ring made a direct and water-bridged hydrogen bonding interaction with the residue Asp-274, which persisted for 41 and 36% of the simulation time. The carbonyl (ketone) functionality in the quinoline-2-one ring demonstrated a water-bridged hydrogen bonding interaction that persisted for 52% of the interactions. Trp-80, the principal residue in the allosteric site of Akt-1, was demonstrated to make a hydrophobic interaction with the ligand. The residues, Asp-274, Asp-292, Gln-79 and Tyr-272, had frequent contact with the ligand throughout the simulation. The RMSD plot of indacaterol shows peaks of around ~ 0.941 Å. The radius of gyration measured for the extendedness of the ligand, equivalent to its moment of inertia, shows peaks of around 5.471 Å. The MolSA was between 376.767 and 389.984 Å. The SASA and PSA ranged from 3.858 to 99.056 and 159.178 to 177.26 Å, respectively.

**Figure 6:**
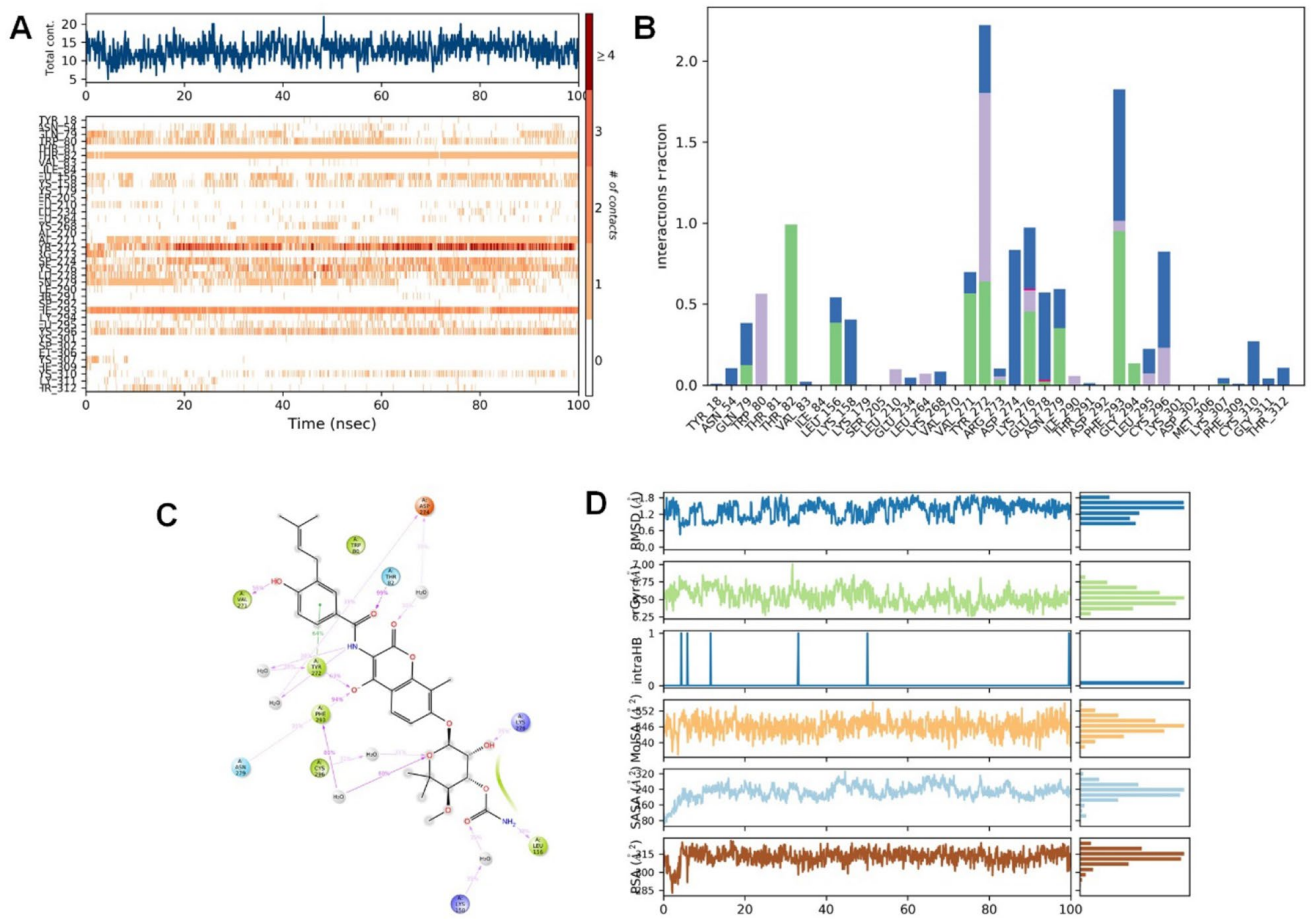
Protein interactions with the novobiocin monitored during the course of trajectory (**A**) Timeline illustration of total specific contacts that several interacting residues makes with a ligand (**B**) Protein-ligand contacts described as histogram (**C**) Various protein- ligand interactions that stays for more than 30.0% of the simulation time (**D**) Various ligand properties in the form of ligand RMSD, radius of gyration (rGyr), molecular surface area (MolSA), solvent‐accessible surface area (SASA) and polar surface (PSA) expressed in Å during the 100 ns of the MD simulation.

Novobiocin: Novobiocin is an antibiotic compound. After the initial fluctuation due to the equilibration for 35 ns, the protein structures RMSD varied between 0.7 to 2.8 Å till the end of the simulation (Fig. 2D). The protein structure fluctuations between 2.1 Å indicated a stable protein structure where the complex had not undergone significant conformational changes. Similarly, the ligand structures RMSD varied between 2.3 and 3.6 Å till the end of the simulation. The ligand structure fluctuations remained between 1.3 Å, indicating that the ligand is stably bound to the kinase allosteric site and has not diffused significantly from the bound position. Figure 6 depicts the ligand–protein interactions as H-bonds, hydrophobic, ionic, and water bridges were observed as protein–ligand contacts recorded throughout the MD simulation over 30% of the simulation time. The hydroxyl group attached to the benzene ring and 3-methylbut-2-en-1-yl ring demonstrated a direct hydrogen bonding interaction with the Val-271 residue that persisted for 56% of the simulation. The aromatic benzene ring demonstrated a π-π interaction with the residue Tyr-272 that persisted for 64% of the simulation. The carbonyl group in the carboxamide linker demonstrated direct hydrogen bonding interaction with the residue Thr-82, which persisted for 99% of the simulation. The nitrogen atom in the carboxamide linker demonstrated two water-bridged interactions with residues Tyr-272 and Asp-274, which persisted for 38 and 39% of the simulations. The hydroxyl group in the oxochromen ring demonstrated a direct hydrogen bonding interaction with Tyr-272, Asn-279 and Phe-293 residues that persisted for 63, 35 and 94% of the simulations. The carbonyl ring of the oxochromen ring demonstrated a water-bridged interaction with the residue Asp-274 that persisted for 30% of the simulations. The 3-methoxy-2,2-dimethyloxan-4-yl carbamate tail in the structure of novobiocin also demonstrated several molecular interactions. The free amino functional group in the carbamate tail demonstrated direct hydrogen bonding interactions with residue, Leu-156, that persisted for 38% of the simulation. The carbonyl functional group in the carbamate tail also demonstrated a water-bridged hydrogen bonding interaction with the Lys-158 residue, which persisted for 35% of the simulations. The hydroxyl group attached to 3-methoxy-2,2-dimethyloxan-4-yl carbamate demonstrated a direct hydrogen bonding interaction with the Lys-276 residue, which persisted for 35% of the simulations. The heterocyclic oxygen atom in the oxan ring demonstrated a water-bridged interaction with Phe-293 residue, which persisted for 80% of the simulations. Phe-293, Thr-82, and Tyr-272 residues had frequent contact with the ligand throughout the simulation. The RMSD plot of novobiocin shows peaks of around ~ 1.907 Å. The radius of gyration measured for the extendedness of the ligand, equivalent to its moment of inertia, shows peaks of around 7.007 Å. The MolSA was around 535.472 to 555.268 Å. The SASA and PSA ranged from 62.237 to 330.887 and 282.819 to 326.224, respectively Å.

Carrella et al., 2016, have attempted to repurpose FDA approved drug to target computational repurposing for oncogenic PI3K/AKT/P70S6K-dependent pathways with the help of network pharmacology and shortlisted niclosamide and pyrvinium pamoate as the potential drugs for for cancer treatment^47^. Carrella et al., 2016 study was not for a specific type of cancer and specific target. In this work, we have specified the target Akt-1 for drug repurposing in NSCLC. In summary, docking the FDA-approved drugs to the allosteric site of Akt-1, followed by MD simulations, allowed us to shortlist the top-14 molecules. The literature on these top-14 molecules suggests that using these in the treatment has added benefits in lung cancer. Vilazodone is developed as an antidepressant drug^48^ which might reduce anxiety for patients with lung cancer. In addition, recently, it has been shown to inhibit the human colon cancer cells HCT116^49^. Vilazodone was srecognized as an inositol polyphosphate multikinase (IPMK) antagonist in a structure-based virtual screening of licenced drugs. Vilazodone inhibited IPMK and Akt phosphorylation^49^, hence, it can be repurposed in lung cancer. The drug indacaterol is a long-acting β_2_-agonists used to treat asthma and COPD^50^. It was recently studied on human lung cancer cells (NSCLC) with EGFR T790M mutation and was found to induce apoptosis in lung cancer cells via downregulation of activated Akt ^51^. In this study, we propose that indacaterol inhibits Akt-1, a newer mechanism of action as an anti-cancer agent. Pitavastatin is a lipid-lowering agent with multifaceted pharmacological properties^52^. This drug is currently the centre of attraction for development as an anti-cancer agent for many different human cancers, and has demonstrated anInhibition of prenylation-dependent Ras/Raf/MEK and PI3K/Akt/mTOR signalling in lung cancer cells and human lung tumour-associated endothelial cells^53^. Moreover, arecent clinical study found it beneficial in combination with neoadjuvant therapy^54^. Nomegestrol is developed as a progestin contraceptive and postmenopausal hormone replacement therapy. However, it is also reported to be anti-cancer in human endometrial RL95-2 cancer cells, both in vitro and in vivo xenograft model^55^. Studies suggest that synthetic progestin medroxyprogesterone acetate (MPA), connected to nomegesterol, can offset oestrogen's positive impact on endothelial function. MPA reduced nitric oxide (NO) production by vascular endothelium in human endothelial cells via inhibition of Akt and ERK signalling cascade^56^. Most recent studies also reveal that progesterone receptor membrane component 1 (PGRMC1) is the prospective therapeutic target in lung cancer. Hence nomegestrol can be repurposed^57^. Raltitrexed was developed as a novel thymidylate synthase inhibitor to treat cancer. It was also reported to be active in human lung cancer cells A549 and human colon cancer cells HCT‐116 in preclinical studies^58^. Raltitrexed improved the anti-cancer outcomes of lapatinib on human oesophageal squamous carcinoma cells by decreasing the phosphorylation of Akt and Erk^59^. Our present study demonstrated that it also possesses allosteric Akt-1 inhibition and is shypothesized to inhibit Akt phosphorylation. Novobiocin was developed and approved as an antibiotic targeting DNA gyrase to treat severe infections due to *Staphylococcus aureus*. However, because of safety or effectiveness concerns, the drug was withdrawn as antibiotics in 2009. Novobiocin was found to hinder the interaction between the CH1 region of p300/CBP and HIF-1α C-TAD, which resulted in down-regulated expression of genes regulated by HIF-1α, especially CA9, resulting in an inhibitory effect on tumour cell proliferation, that eventually decreased mRNA expression of AKT1 and mTOR in A549 and MCF-7 cells^60^. Further, novobiocin has been tried in several cancers and highly explored by medicinal chemists as derivatives and found beneficial in multiple human cancers^61^. Ezetimibe is a lipid-lowering drug currently used in therapy and was found to be inhibiting the growth of gastrointestinal cancer^62^. Ditazole was developed as an analgesic and anti-inflammatory drug similar to NSAIDs with additional antiplatelet properties^63^. Bone is among the most frequent site of metastasis for all malignancies. Bone metastases occur in up to 75% of patients with advanced breast and prostate cancer and up to 40% of patients with advanced lung cancer^64^. Palliative radiation or narcotic analgesic medications are prescribed for bone pain. Hypercalcemia, nerve compression, pathological fracture, spinal cord compression, and orthopaedic surgical complications could arise from these can harm a person's quality of life (QoL)^65^. These properties will have additional benefits in relieving cancer-induced pain/ inflammation. Nebivolol is a cardiovascular drug, β-blocker and recently found inhibition of mitochondrial activity, thereby inhibiting angiogenesis and arresting human tumour growth^66^. According to recent research, prior β-blocker usage is related to prolonged time-to-discontinuation (TTD) and survival rates (OS) in treatment-naive patients who have advanced lung cancer following first-line EGFR-TKIs^67^. Floxuridine is also known as 5-fluorodeoxyuridine, a pyrimidine analogue. This molecule was developed as an antimetabolite/anti-cancer agent used to treat colorectal cancer^68^. However, its ability to bind Akt-1 allosteric site was not being discovered to date. Delorazepam is a metabolite of diazepam and was developed as an antidepressant drug to reduce anxiety also therapeutically used in bipolar disorders^69^. The reduction of anxiety is necessary for patients with cancer. Valganciclovir is a purine metabolite developed as an antiviral agent to treat human cytomegalovirus. Valganciclovir is a l-valyl ester of ganciclovir, a prodrug whose concentrations after oral administration are low and transient, t-half is 30 min^70,71^. It was recently found to be an anti-cancer agent. Several clinical studies have found it beneficial in chemotherapy in combination, especially for treating glioblastoma^72^. Hence, targeting the drug to the lung as an appropriate pharmaceutical formulation technology could help repurpising and developing valganciclovir as a drug in NSCLC. The drug dasatinib was developed as a tyrosine kinase inhibitor (TKIs) and used to treat different human cancer. Song et al.^73^ demonstrated that dasatinib exerted growth inhibitory activity in apoptosis in gefitinib-sensitive EGFR mutant lung cancer cells in vitro via down-regulation of activated survival proteins, namely Akt and STAT3. Recently proven, its effect in lung cancer cells^74^. Here we are adding the novel mechanism of action for dasatinib as an allosteric Akt-1 inhibitor. Lorazepam is an anti-anxiety drug similar to diazepam. This drug is found beneficial in chemotherapy-induced emesis and anxiety^75^. Thus repurposing these drugs could have added advantages in lung cancer.

The protein–ligand RMSD plot generated after the MD simulation and various other aspects like protein–ligand contacts, ligand–protein contacts, various ligand properties like ligand RMSD radius of gyration (rgyr), molecular surface area (MolSA), solvent accessible surface area (SASA), and polar surface area (PSA). Analysis of these parameters also demonstrated a stable binding for the dasatinib, valganciclovir, indacaterol and novobiocin within the allosteric binding pocket of Akt-1. The floxuridine, delorazepam, ezetimibe and pitavastatin had moderate binding stability, and the other drugs had higher than 3.0 Å fluctuations. Hence, only valganciclovir, dasatinib, indacaterol and novobiocin are further studied for computational efficacy and toxicity predictions.

### Prediction of toxicity, cell line cytotoxicity and biological activity prediction

ProTox-II server classifies the chemicals into different classes based on the predicted LD50. ProTox-II classifies chemicals into toxicity classes sper the globally harmonized system of classification of labelling of chemicals (GHS). Class I belongs to fatal if swallowed (LD50 ≤ 5), and Class II is fatal if swallowed with a higher LD50 range (5 < LD50 ≤ 50). Similarly, Class III is toxic if swallowed (50 < LD50 ≤ 300), Class IV is harmful if swallowed (300 < LD50 ≤ 2000), Class V may be harmful if swallowed (2000 < LD50 ≤ 5000), and Class VI is said to be non-toxic (LD50 > 5000). The results in our studies from the ProTox-II server, valganciclovir,were categorized as class V. In contrast, dasatinib, indacaterol and novobiocin belonged to class IV, with LD50 range from 369 to 5000 mg/kg. Indacaterol belonged to class IV and was harmful if swallowed. Further, valganciclovir and dasatinib predicted a high risk of carcinogenicity, and not with indacaterol and novobiocin. Further, dasatinib, indacaterol and novobiocin were predicted to be immunotoxic. The results are represented in Table 4.

**Table 4:** Results of oral toxicity prediction for shortlisted compounds.

Endpoint	Target	Valganciclovir	Dasatinib		Indacaterol		Novobiocin	
Organ toxicity and Toxicity Endpoints	Hepatotoxicity	Inactive/0.92	Inactive/0.63		Inactive/ 0.74		Inactive/ 0.58	
	Carcinogenicity	Active/0.50	Active/0.50		Inactive/ 0.60		Inactive/ 0.66	
	Immunotoxicity	Inactive/0.99	Active/0.66		Active/ 0.80		Active/ 0.99	
	Mutagenicity	Inactive/0.68	Inactive/0.67		Inactive/ 0.61		Inactive/ 0.56	
	Cytotoxicity	Inactive/0.74	Inactive/0.63		Inactive/ 0.66		Inactive/ 0.56	
	LD50 (mg/kg)	5000	1000		369		962	
	Toxicity class	5	4		4		4	
Tox21-Nuclear receptor signalling pathways (Prediction/Probability)	Aryl hydrocarbon Receptor (AhR)	Inactive/0.97	Inactive/0.86		Inactive/ 0.76		Inactive/ 0.84	
	Androgen Receptor (AR)	Inactive/0.99	Inactive/0.98		Inactive/ 0.97		Inactive/ 0.97	
	Androgen Receptor Ligand Binding Domain (AR-LBD)	Inactive/0.99	Inactive/0.95		Inactive/ 0.94		Inactive/ 0.96	
	Aromatase	Inactive/0.98	Inactive/0.89		Inactive/ 0.83		Inactive/ 0.91	
	Estrogen Receptor Alpha (ER)	Inactive/0.98	Inactive/0.87		Inactive/ 0.84		Inactive/ 0.90	
	Estrogen Receptor Ligand Binding Domain (ER-LBD)	Inactive/0.99	Inactive/0.99		Inactive/ 0.96		Inactive/ 0.96	
	Peroxisome Proliferator-Activated Receptor Gamma (PPAR-Gamma)	Inactive/0.99	Inactive/0.83		Inactive/ 0.95		Inactive/ 0.93	
Tox21-Stress response pathway	Nuclear factor (erythroid-derived 2)-like 2/antioxidant responsive element (nrf2/ARE)	Inactive/0.99	Inactive/0.96		Inactive/ 0.94		Inactive/ 0.95	
	Heat shock factor response element (HSE)	Inactive/0.98	Inactive/0.96		Inactive/ 0.94		Inactive/ 0.95	
	Mitochondrial Membrane Potential (MMP)	Inactive/0.98	Inactive/0.82		Inactive/ 0.80		Inactive/ 0.68	
	Phosphoprotein (Tumor Suppressor) p53	Inactive/0.96	Inactive/0.96		Inactive/ 0.85		Inactive 0.74/	
	ATPase family AAA domain containing protein 5 (ATAD5)	Inactive/0.97	Inactive/0.96		Inactive/ 0.88		Inactive/ 0.88	
Fathead minnow LC50 (96 h)	mg/ml	40.54 (MoA = Narcosis)	N/A (Narcosis)		23.18 (Narcosis)		N/A (AChE inhibition)	
48-h Daphnia magna LC50	mg/L	39.44 (Single model)	159.42		3.03		0.0303	
48-h T.pyriformis IGC50	mg/ml	23.06 (Nearest neighbour)	4.63		2.20		15.63	
Developmental toxicity	Value	Developmental toxicant Predvalue = 0.73	Developmental toxicant Predvalue = 0.74		Developmental toxicant Predvalue = 0.74		Developmental toxicant Predvalue = 0.92	
Mutagenicity	Result	Mutagenicity negative Predvalue = 0.31	Mutagenicity negative Predvalue = 0.39		Mutagenicity negative Predvalue = 0.03		Mutagenicity negative Predvalue = 0.55	
Oral rat LD50	mg/kg	N/A (Consensus)	1383.10		776.61		1188.43	
Bioconcentration factor	Value	0.11 (Consensus)	3.51		7.29		N/A	

Properties include organ toxicity, toxicity endpoints, Tox21-Nuclear receptor signalling pathways, Tox21-stress response pathway, fathead minnow LC50 (96 h), developmental toxicity, oral rat LD50, bioaccumulation factor of the shortlisted four compounds in the table. These data are generated from ProTox-II server-based prediction tools.

CLC-Pred is a predictor tool for cell line cytotoxicity and reduces the time and cost of the experimental in vivo screening, especially for anti-cancer activity^37^. In this tool the, cytotoxicity prediction was done based on Naive Bayes learning algorithms. The structure-cell line toxicity relationship was assessed using particular training sets with a leave-one-out cross-validation procedure^76^. The prediction of cytotoxicity was made in terms of Pa (probable activity) and Pi (probable inactivity), which ranged from 0.000 to 1.000. By default in PASS, the Pa = Pi value is considered a threshold; those with Pa > Pi are proposed to be cytotoxic or active, wherein Pa > 0.5 is considered highly cytotoxic and highly active, Pa > 0.3 is believed to have intermediate activity, and Pa < 0.3 is deemed to be the lowest activity^37,77,78^. The in silico prediction of cytotoxic activity using CLC-Pred for four compounds, valganciclovir, dasatinib, indacaterol and novobiocin, with Pa > Pi for various lung cancer cell lines, are provided in Table 5. The results indicate that valganciclovir is expected to exhibit more cytotoxic potential against NSCLC cells NCI-H1299 (Pa = 0.254, and Pi = 0.115). However, this molecule was predicted to be cytotoxic against non-tumorigenic lung fibroblasts, i.e. MRC5 cells (Pa = 0.388, Pi = 0.025). Prediction from the PASS online server using the 2D structure based on their mechanisms of action depending on their interaction with targets and general effects such as antineoplastic, antihypertensive, antiepileptic etc.

**Table 5:** Cytotoxicity prediction of the shortlisted compounds on tumour and non-tumour cell lines of the lung using CLC-Pred at Pa > Pi.

Tumour cell	Pa	Pi	Non-tumour cell line	Pa	Pi
**Valganciclovir**					
NCI-H1299	0.254	0.115	MRC5	0.388	0.025
EKVX	0.234	0.098	–	–	–
NCI-H23	0.238	0.151	–	–	–
NCI-H322M	0.226	0.207	–	–	–
**Dasatinib**					
NCI-H23	0.306	0.100	–	–	–
HOP-92	0.263	0.129	–	–	–
HOP-62	0.189	0.144	–	–	–
**Indacaterol**					
DMS-114	0.597	0.010	–	–	–
NCI-H187	0.496	0.015	–	–	–
NCI-H69	0.261	0.063	–	–	–
PC-6	0.290	0.096	–	–	–
HOP-62	0.245	0.088	–	–	–
NCI-H838	0.324	0.228	–	–	–
NCI-H23	0.226	0.165	–	–	–
NCI-H128	0.132	0.072	–	–	–
**Novobiocin**					
NCI-H187	0.395	0.075	WI-38 VA13	0.177	0.123
NCI-H838	0.416	0.116	–	–	–
SHP77	0.156	0.099	–	–	–
SW1573	0.153	0.145	–	–	–

The data are predicted values and are obtained from the CLC-Pred web-based prediction tool.

The biological activity spectrum refers to the list of biological activities a chemical substance exhibits due to its interaction with different biological entities^38^. In drug repurposing, re-tasking, or re-profiling, new uses for existing or sauthorized medications approved or clinically unrelated or original medicinal indication are established ^79^. The anti-carcinogenic activity was predicted for molecules valganciclovir (Pa = 0.509, Pi = 0.018), indacaterol (Pa = 0.342, Pi = 0.025), and novobiocin (Pa = 0.52, Pi = 0.017). The different predicted results are illustrated in Table 6.

**Table 6:** The predicted biological effect by the four shortlisted compounds using PASSOnline webserver at Pa > Pi.

Predicted biological activities	Pa	Pi
**Valganciclovir**		
Immunostimulant	0.9	0.004
Aldehyde oxidase substrate	0.872	0.002
Antiviral (Herpes)	0.861	0.002
Antiviral	0.809	0.004
DNA-directed DNA polymerase inhibitor	0.785	0.003
Radiosensitizer	0.77	0.004
Antiinfective	0.77	0.005
Cytostatic	0.763	0.008
Immunomodulator	0.749	0.003
Antiviral (CMV)	0.698	0.001
Antiviral (Poxvirus)	0.662	0.011
ADP-thymidine kinase inhibitor	0.664	0.017
Antineoplastic antimetabolite	0.652	0.005
Fibroblast growth factor agonist	0.651	0.012
Macrophage stimulant	0.639	0.005
Immunosuppressant	0.641	0.024
Chemosensitizer	0.591	0.007
Undecaprenyl-phosphate mannosyltransferase inhibitor	0.593	0.011
DNA synthesis inhibitor	0.589	0.011
Peptide agonist	0.601	0.028
DNA polymerase I inhibitor	0.557	0.005
Mannotetraose 2-alpha-N-acetylglucosaminyltransferase inhibitor	0.594	0.045
Thiol oxidase inhibitor	0.555	0.016
Xanthine oxidase substrate	0.528	0.002
Anticarcinogenic	0.509	0.018
**Dasatinib**		
Tyrosine kinase inhibitor	0.825	0.004
Lck kinase inhibitor	0.802	0.002
Src kinase inhibitor	0.794	0.003
Protein kinase inhibitor	0.713	0.007
Proto-oncogene tyrosine-protein kinase Fyn inhibitor	0.694	0.003
Proto-oncogene tyrosine-protein kinase Fgr inhibitor	0.687	0.002
T cell inhibitor	0.604	0.003
Signal transduction pathways inhibitor	0.569	0.022
Protein-tyrosine kinase Lyn inhibitor	0.53	0.002
Immunosuppressant	0.555	0.034
Growth factor agonist	0.511	0.004
Protein-tyrosine kinase p55(blk) inhibitor	0.506	0.003
Anaphylatoxin receptor antagonist	0.55	0.05
**Indacaterol**		
Adrenergic	0.813	0.003
Chronic obstructive pulmonary disease treatment	0.717	0.004
Bronchodilator	0.661	0.003
Antiasthmatic	0.667	0.01
Adrenaline agonist	0.655	0.002
Proteasome ATPase inhibitor	0.662	0.02
Muramoyltetrapeptide carboxypeptidase inhibitor	0.642	0.02
Glyceryl-ether monooxygenase inhibitor	0.558	0.039
Beta adrenoreceptor agonist	0.479	0.002
Spasmolytic	0.488	0.03
Uterine relaxant	0.462	0.007
Mycothiol-S-conjugate amidase inhibitor	0.449	0.034
HERG 1 channel blocker	0.448	0.035
UGT2B12 substrate	0.441	0.034
Mannotetraose 2-alpha-N-acetylglucosaminyltransferase inhibitor	0.479	0.076
Beta 1 adrenoreceptor agonist	0.395	0.003
Antidiabetic	0.387	0.047
Skeletal muscle relaxant	0.363	0.04
CYP2J2 substrate	0.451	0.13
Antineoplastic alkaloid	0.342	0.025
**Novobiocin**		
Antimycobacterial	0.77	0.004
Antibacterial	0.69	0.005
Antineoplastic	0.69	0.028
Lactase inhibitor	0.629	0.01
DNA gyrase inhibitor	0.524	0.001
HIF1A expression inhibitor	0.562	0.04
Anticarcinogenic	0.526	0.017
CYP2H substrate	0.567	0.083

Data presented are predicted values from the web-server-based tool PASSOnline.

## Conclusion

In this computational study, we identified eight best hits that could bind to the Akt-1 allosteric site, capable of treating NSCLC. The top best hits are valganciclovir, dasatinib, indacaterol, novobiocin, ezetimibe, delerozepam, pitavastatin and nebivolol. These compounds could be repurposed as potent Akt-1 allosteric inhibitors capable of binding directly to the site where the co-crystal ligand, in a similar binding mode as standard ligand 12j or 0R4, thus it could stabilize the 'PH-in' form of the Akt-1. Further, these compounds are predicted to be cytotoxic on human lung cancer cells, and valganciclovir was the best anti-cancer agent among the tested compounds. Thus, the shortlisted molecules could represent novel treatment options and form the basis for further experimental validation and step to rational drug design of a new class of allosteric Akt-1 inhibitors.

## Supplementary Information

Supplementary Information 1.

Supplementary Information 2.

## Data Availability

Most of the data used is mentioned in the manuscript. The data is also supplemented with the manuscript.
