# Supplementary material for: Computational drug repurposing of Akt-1 allosteric inhibitors for non-small cell lung cancer
Source: Sci Rep. 2023 May 16;13:7947. doi: 10.1038/s41598-023-35122-7 (PMC10188557; doi:10.1038/s41598-023-35122-7)
Supplement: Supplementary file 1 — Supplementary Information 1. [file 41598_2023_35122_MOESM1_ESM.pdf]

Supplementary Data

Computational Drug Repurposing of Akt-1 Allosteric Inhibitors for Non-Small Cell Lung Cancer

Krishnaprasad Baby[1], Swastika Maity[1], Chetan Hasmukh Mehta[2], Usha Y. Nayak[2], Gautham G. Shenoy[3], Karkala Sreedhara Ranganath Pai[1], Kuzhuvelil B. Harikumar[4], Yogendra Nayak[1*]

[1]Department of Pharmacology, Manipal College of Pharmaceutical Sciences, Manipal Academy of Higher Education, Manipal, 576104, Karnataka, India

[2]Department of Pharmaceutics, Manipal College of Pharmaceutical Sciences, Manipal Academy of Higher Education, Manipal, 576104, Karnataka, India

[3]Department of Pharmaceutical Chemistry, Manipal College of Pharmaceutical Sciences, Manipal Academy of Higher Education, Madhav Nagar, Manipal, 576 104, Karnataka, India

[4]Cancer Research Program, Rajiv Gandhi Centre for Biotechnology (RGCB), Thiruvananthapuram 695014, India

Corresponding Author: Dr Yogendra Nayak, Associate Professor, Department of Pharmacology, Manipal College of Pharmaceutical Sciences, Manipal Academy of Higher Education, Manipal, Karnataka, 576104, India; e-mail: yogendra.nayak@manipal.edu; Mob: +919448154003; ORCID: https://orcid.org/0000-0002-0508-1394

3D interaction data

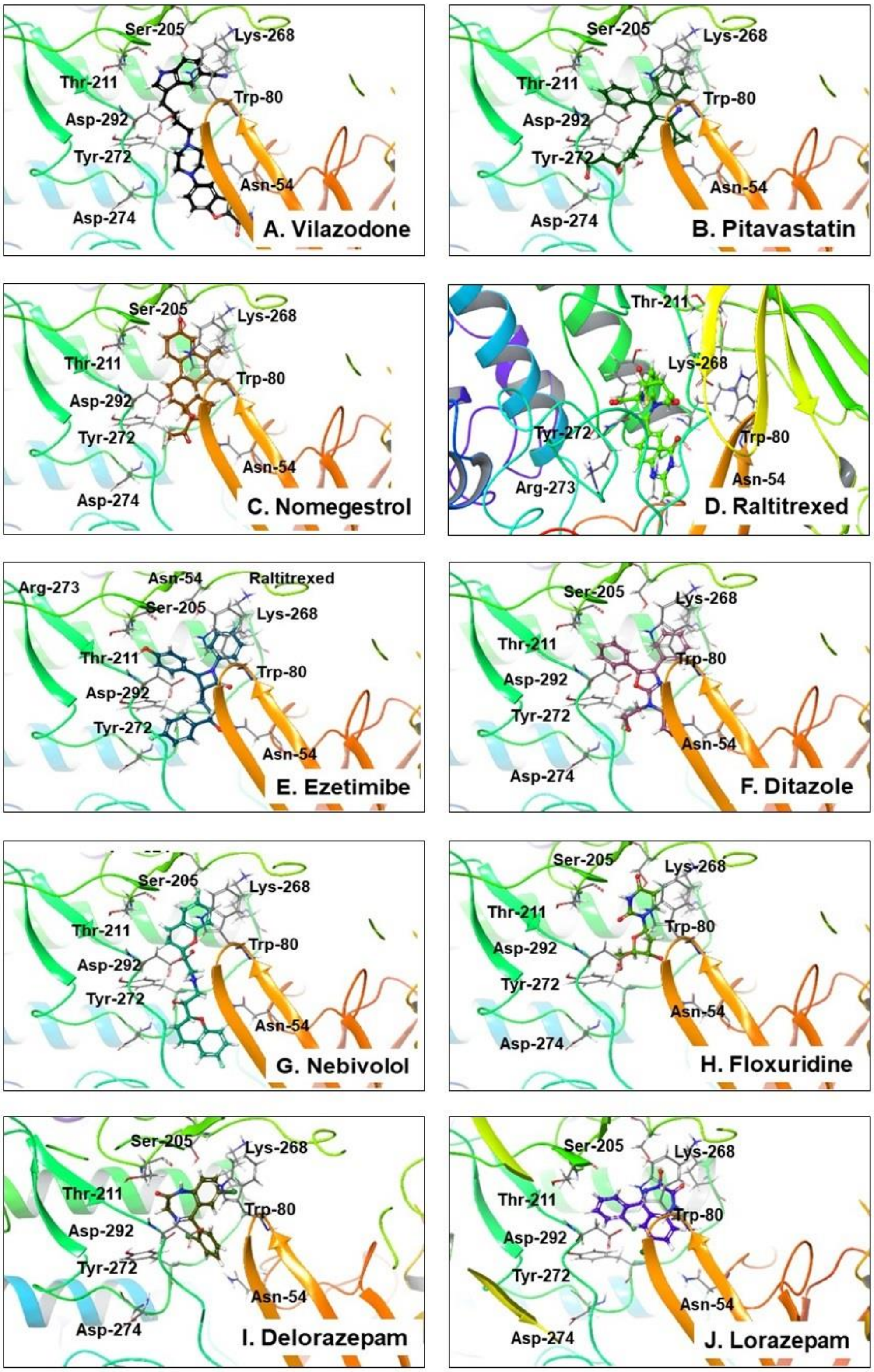

Fig 1S: Ligand-protein interactions displayed by molecules with in the Akt-1 allosteric site (PDB ID: 4EJN) in 3D

MD simulation data

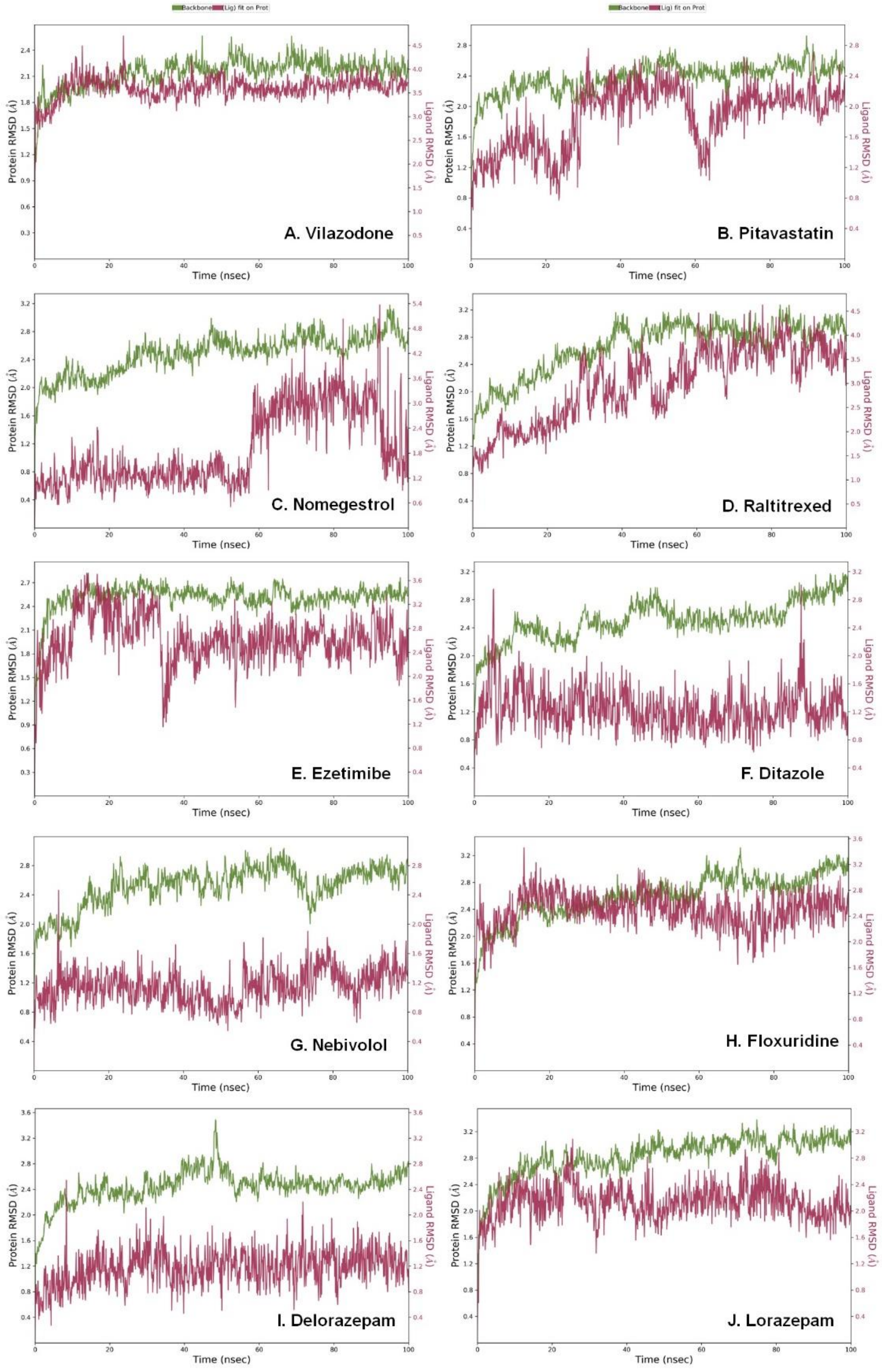

Fig 2S. Root Mean Square Deviation (RMSD) observed during MD simulation molecules for a specified frame relative to a reference frame.

Vilazodone is an antidepressant medication. After the initial fluctuation due to the equilibration for 35 ns, the protein structures RMSD varied between 0.6 Å to 2.3 Å till the end of the simulation (**Fig 2S.A**). The protein structure fluctuations between 1.7 Å indicated a stable protein structure where the complex has not undergone significant conformational changes. Similarly, the ligand structures RMSD varied between 1.0 Å to 3.8 Å till the end of the simulation. The ligand structure fluctuations remained between 2.8 Å, indicating that the ligand is stably bound to the kinase allosteric site and has not diffused significantly from the bound position. **Fig 3S** depicts the ligand-protein interactions as H-bonds, hydrophobic, ionic, and water bridges were observed as protein-ligand contacts recorded throughout the MD simulation over 30% of the simulation time. The benzene ring in the indole moiety of vilazodone demonstrated a π-π interaction with the Trp-80 residue, which persisted for 63% of the simulation time. The cyano group attached at position-5 of the indole ring demonstrated a direct hydrogen bonding interaction with Thr-211 residue. The same functionality demonstrated a water-bridged hydrogen bonding interaction with Tyr-272 residue, which persisted for 51% of the simulations. The piperazine ring in vilazodone demonstrated a π-cation and direct hydrogen bonding interaction with Tyr-272 residue. The 2-carboxamide chain in vilazodone demonstrated several direct and water-bridged hydrogen bonding interactions with residues. The carbonyl part of the carboxamide chain demonstrated a water-bridged hydrogen bonding interaction with Glu-85 residue, which persisted for 83% of simulations. The same functionality demonstrated water-bridged hydrogen bonding interactions, which persisted for 43% of the simulations. The carboxamide functional group also made another water-bridged hydrogen bonding interaction, which persisted for 44% of the simulations. The amide part of carboxamide moiety demonstrated water-bridged hydrogen bonding interactions with Glu-85, and Glu-298, which persisted for 70%, and 61% of the simulations. The same moiety demonstrated a direct hydrogen bonding interaction with Cys-296 residue, which persisted for 87% of the simulations. The Cys-310 residue demonstrated a hydrophobic contact with the ligand. The residues, Glu-85, Tyr-272, Glu-298 and Cys-296, demonstrated frequent contact with the ligand throughout the simulation. The RMSD plot of vilazodone shows peaks of around ~2.7 Å. The radius of gyration measured for the extendedness of the ligand, equivalent to its moment of inertia, shows peaks of around 6.867 Å. The MolSA was around 422.637-437.314 Å. The SASA and PSA ranged from 21.755 to 101.185 and 202.101 to 215.797 Å, respectively.

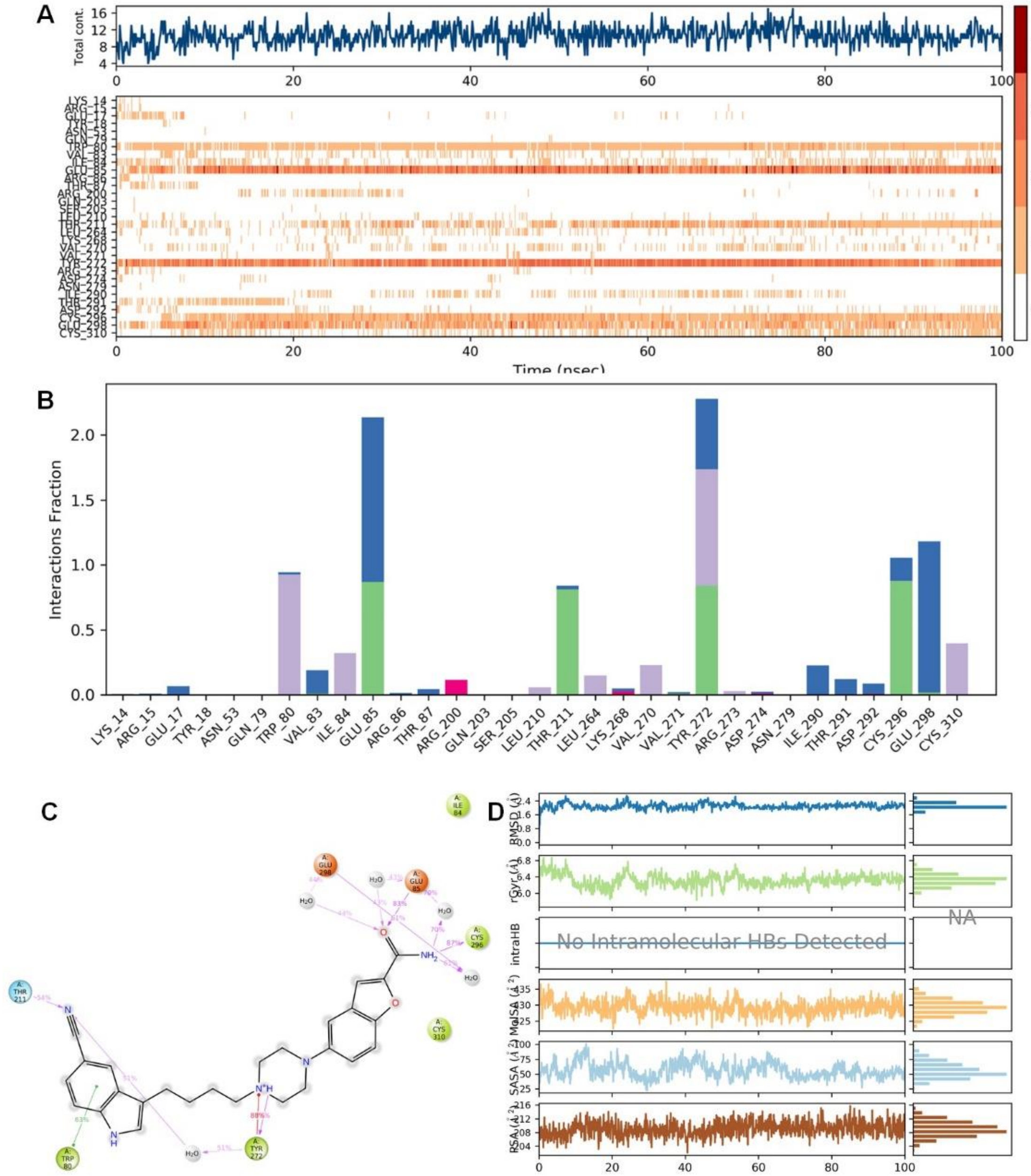

Fig 3S. Protein interactions with the vilazodone monitored during the course of trajectory (A) Timeline illustration of total specific contacts that several interacting residues makes with a ligand (B) Protein-ligand contacts described as histogram (C) Various protein-ligand interactions that stays for more than 30.0% of the simulation time (D) Various ligand properties in the form of ligand RMSD, radius of gyration (rGyr), molecular surface area (MolSA), solvent-accessible surface area (SASA) and polar surface (PSA) expressed in Å during the 100 ns of the MD simulation

Pitavastatin is a statin or cholesterol-lowering agent. After the initial fluctuation due to the equilibration for 35 ns, the protein structures RMSD varied between 0.8 Å to 2.5 Å till the end

of the simulation (**Fig 2S.B**). The protein structure fluctuations between 1.7 Å indicated a stable protein structure where the complex has not undergone significant conformational changes. Similarly, the ligand structures RMSD varied between 1.75 Å to 2.4 Å till the end of the simulation. The ligand structure fluctuations remained between 0.65 Å, indicating that the ligand is stably bound to the kinase allosteric site and has not diffused significantly from the bound position. **Fig 4S** depicts the ligand-protein interactions as H-bonds, hydrophobic, ionic, and water bridges were observed as protein-ligand contacts recorded throughout the MD simulation over 30% of the simulation time. The 4-fluorophenyl ring demonstrated a π-π interaction with the residue, Trp-80, that persisted for 87% of the simulation. The N-1 of the quinoline ring of pitavastatin demonstrated a direct hydrogen bonding interaction with the Gln-79 residue that persisted for 41% of the simulation. The hydroxyl groups attached at positions 3 and 5- of the dihydroxyhept-6-enoic acid side chain demonstrated hydrogen bonding interactions during simulation. The hydroxyl group at position-3 of the dihydroxyhept-6-enoic acid side chain demonstrated a direct hydrogen bonding interaction with the Asp-274 residue, which persisted for 62% of the simulation time. The hydroxyl group at position-4 of the dihydroxyhept-6-enoic acid side chain demonstrated a water-bridged hydrogen bonding interaction with the Asp-271 residue, which persisted for 78% of the simulations. The hydroxyl group of the carboxyl functional group of the dihydroxyhept-6-enoic acid side chain demonstrated a water-bridged hydrogen bonding interaction with the Asp-292d residue, which persisted for 74% of the simulations. The functionality demonstrated a direct hydrogen bonding interaction and a salt bridge interaction with Lys-276 residue, which persisted for 45 and 49% of the simulation. The same residues Asp-292 demonstrated a water-bridged hydrogen bonding interaction with the (carbonyl –C=O) part of the carboxylic acid, which persisted for 63% of the simulations. The same carbonyl part interacted via a direct hydrogen bonding interaction with Lys-276 residue, which persisted for 50% of the simulations. A hydrophobic interaction was observed with the Tyr-272 and the ligand. The residues, Trp-80, Lys-276, Arg-273, and Asp-292, had frequent contact with the ligand throughout the simulation. The RMSD plot of pitavastatin shows peaks of around ~1.56 Å. The radius of gyration measured for the extendedness of the ligand, equivalent to its moment of inertia, shows peaks of around 4.801 Å. The MolSA was around 380.386 to 397.35 Å. The SASA and PSA ranged from 21.867 to 124.89 and 140.666 to 181.693 Å, respectively.

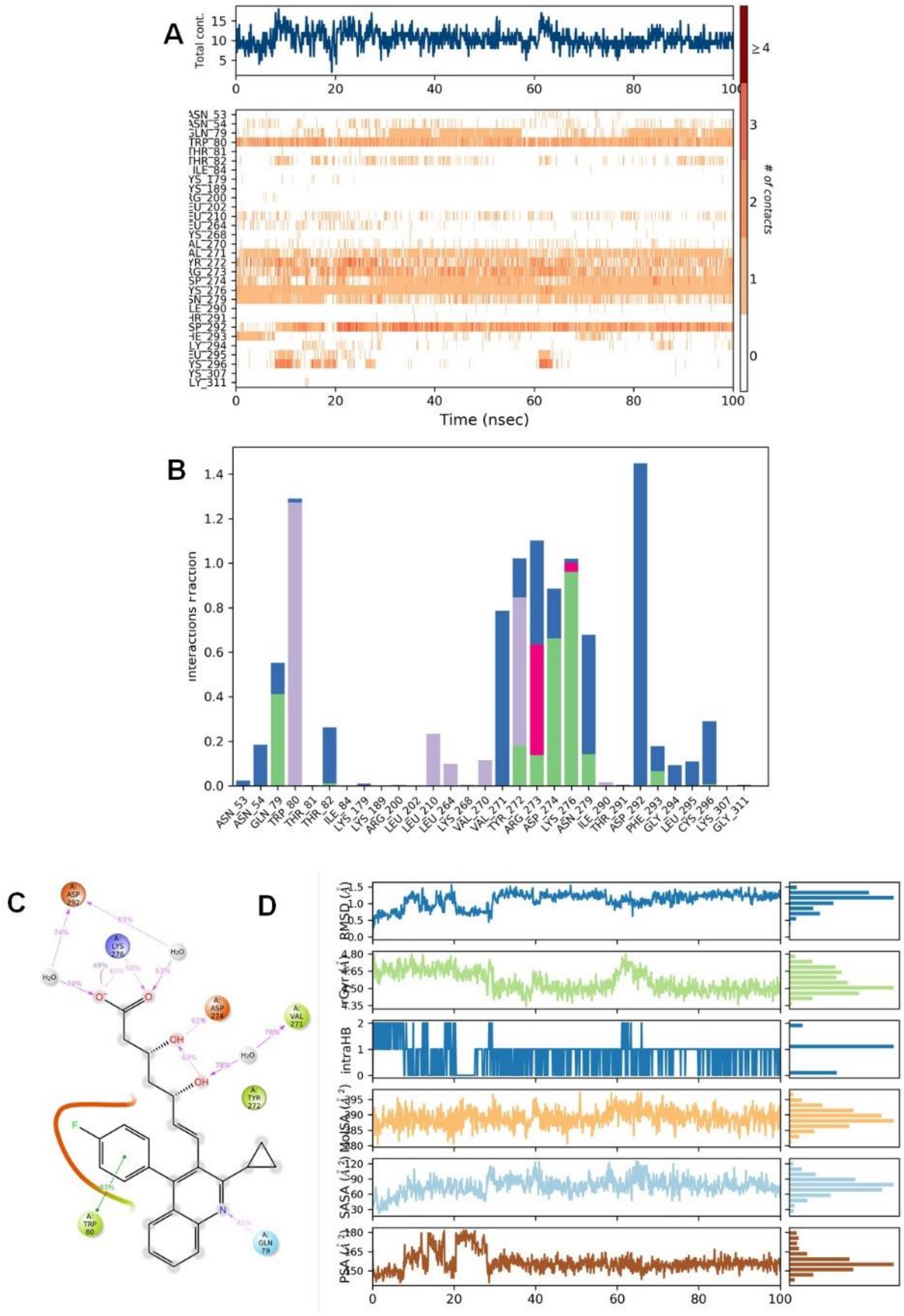

Fig 4S. Protein interactions with the pitavastatin monitored during the course of trajectory (A) Timeline illustration of total specific contacts that several interacting residues makes with a ligand (B) Protein-ligand contacts described as histogram (C) Various protein-ligand interactions that stays for more than 30.0% of the simulation time (D) Various ligand properties in the form of ligand RMSD, radius of gyration (rGyr), molecular surface area (MolSA), solvent-accessible surface area (SASA) and polar surface (PSA) expressed in (Å) during the 100 ns of the MD simulation

Nomegestrol is a drug used for the treatment of gynaecological disorders. Following an initial fluctuation due to the equilibration for 35 ns, the protein structures RMSD varied between 0.8 Å to 2.8 Å till the end of the simulation (**Fig 2S.C**). The protein structure fluctuations between 2.0 Å indicated a low, stable protein structure where the complex has undergone slight significant conformational changes. Similarly, the ligand structures RMSD varied between 2.4 Å to 4.8 Å till the end of the simulation. The ligand structure fluctuations remained between 2.4 Å, indicating that the ligand is bound at the kinase allosteric site and has diffused significantly from its original bound position. **Fig 5S** depicts the ligand-protein interactions as H-bonds, hydrophobic, ionic, and water bridges were observed as protein-ligand contacts recorded throughout the MD simulation over 30% of the simulation time. The terminal acetyl functional group demonstrated a direct hydrogen bonding interaction with the Phe-293 residue that persisted for 65% of the simulations. The 17-hydroxyl functional group of nomegestrol demonstrated direct hydrogen bonding interactions with residues Thr-82 and Asp-292 that persisted for 90 and 99% of the simulations. The residues Trp-80 and Tyr-272 made hydrophobic contacts with the ligand. The residues Thr-82 and Tyr-272 demonstrated frequent contact with the ligand. The RMSD plot of nomegestrol shows peaks of around ~0.77 Å. The radius of gyration measured for the extendedness of the ligand, equivalent to its moment of inertia, shows peaks of around 3.961 Å. The MolSA was around 300.868 to 311.242 Å. The SASA and PSA ranged from 5.995 to 110.471 and 97.823 to 117.473 Å, respectively.

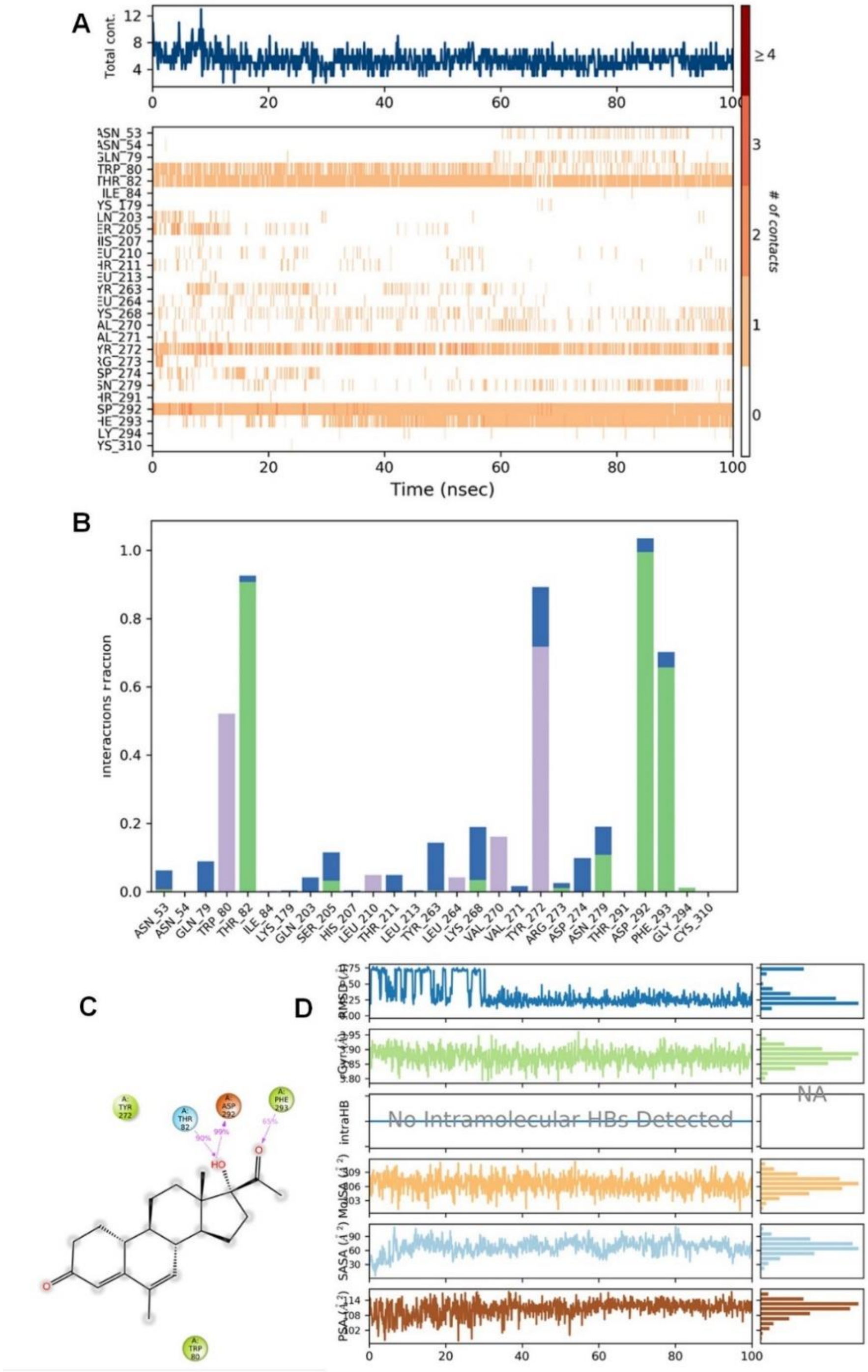

Fig 5S. Protein interactions with the nomegestrol monitored during the course of trajectory (A) Timeline illustration of total specific contacts that several interacting residues makes with a ligand (B) Protein- ligand contacts described as histogram (C) Various protein- ligand interactions that stays for more than 30.0% of the simulation time (D) Various ligand properties in the form of ligand RMSD, radius of gyration (rGyr), molecular surface area (MolSA), solvent-accessible surface area (SASA) and polar surface (PSA) expressed in Å during the 100 ns of the MD simulation

Raltitrexed is a drug used in the treatment of advanced colorectal cancer. Following an initial fluctuation due to the equilibration for 35 ns, the protein structures RMSD varied between 1.0 Å to 2.9 Å till the end of the simulation (**Fig 2S. D**). The protein structure fluctuations between 1.9 Å indicated a stable protein structure where the complex has not undergone significant conformational changes. Similarly, the ligand structures RMSD varied between 2.2 Å to 4.0 Å till the end of the simulation. The ligand structure fluctuations remained between 1.8 Å, indicating that the ligand is stably bound to the kinase allosteric site and has not diffused significantly from the bound position. **Fig 6S** depicts the ligand-protein interactions as H-bonds, hydrophobic, ionic, and water bridges were observed as protein-ligand contacts recorded throughout the MD simulation over 30% of the simulation time. The 4-oxo functional group in the quinazoline ring demonstrated a water-bridged interaction with the residue Glu-17, which persisted for 91% of the simulations. The thiophene ring in Raltirexed demonstrated a π-cation interaction with the residue, Arg-273, which persisted for 37% of the simulations. The 2-carbonyl side group in Raltirexed demonstrated a direct and water-bridged hydrogen bonding interactions with Lys-276, Arg-273, and Asp-274, which persisted for 95, 56 and 58% of the simulations. The carboxyl functional group in Raltirexed demonstrated direct hydrogen bonding interactions with residues, Gly-294, Leu-265 and Cys-296, that persisted for 45, 43, 47 and 49% of the simulations. The residue Lys-307 demonstrated two hydrogen bonding interactions with the terminal carboxylic functional group, which persisted for 39 and 40% of the simulations. Similarly, two direct hydrogen bonding interactions were observed with the same functional group and Gly-311 residue, which persisted for 33 and 35% of the simulations. A water-bridged hydrogen bonding interaction was observed with the hydroxyl group of the terminal carboxyl functional group demonstrated and the ligand that persisted for 32% of the simulations. The residue Trp-80 demonstrated frequent contact with the ligand throughout the simulation. The RMSD plot of raltitrexed shows peaks of around ~ 2.117 Å. The radius of gyration measured for the extendedness of the ligand, equivalent to its moment of inertia, shows peaks of around 6.352 Å. The MolSA was around 400.651 to 415.258 Å. The SASA and PSA ranged from 35.747 to 145.319 and 262.279 to 300.173 Å, respectively.

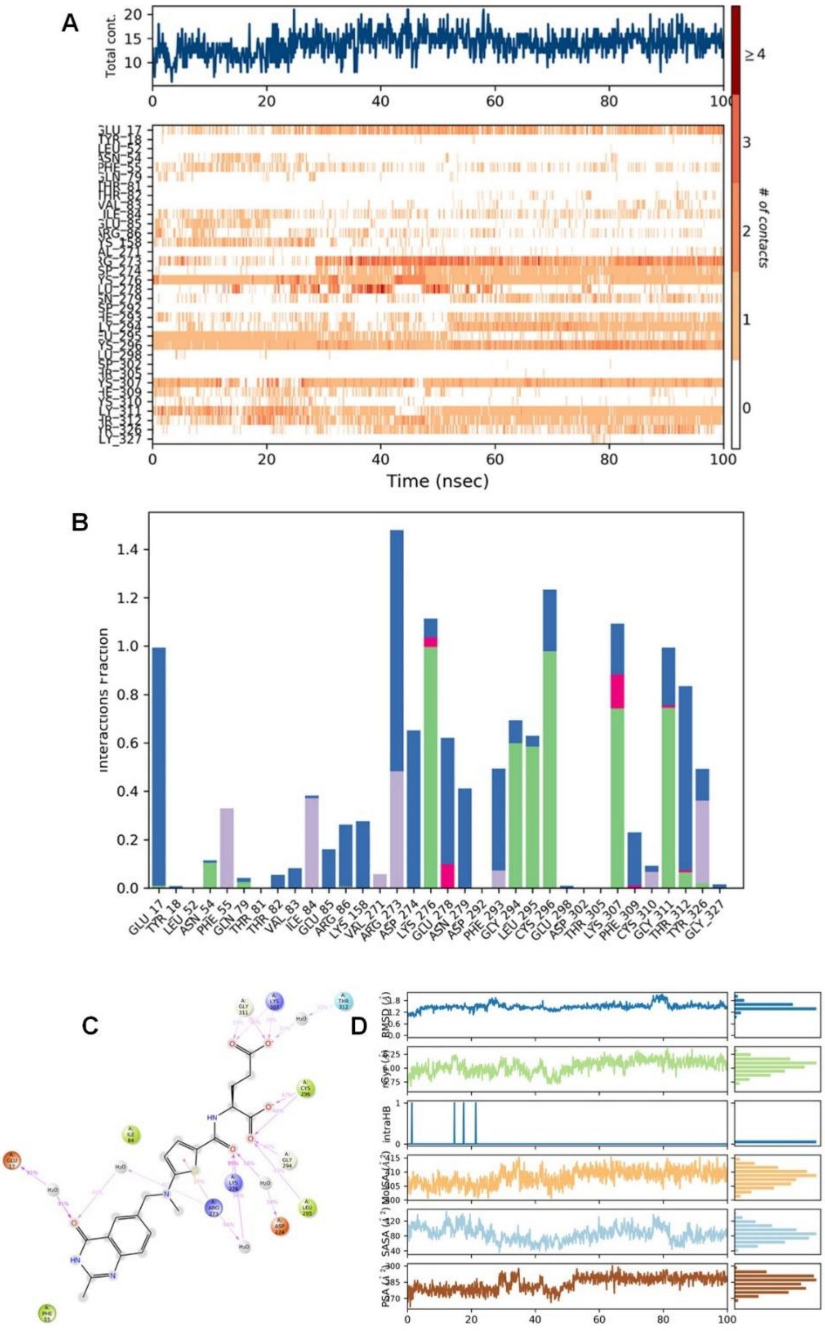

Fig 6S. Protein interactions with the raltitrexed monitored during the course of trajectory (A) Timeline illustration of total specific contacts that several interacting residues makes with a ligand (B) Protein- ligand contacts described as histogram (C) Various protein- ligand interactions that stays for more than 30.0% of the simulation time (D) Various ligand properties in the form of ligand RMSD, radius of gyration (rGyr), molecular surface area (MolSA), solvent-accessible surface area (SASA) and polar surface (PSA) expressed in Å during the 100 ns of the MD simulation

Ezetimibe is a hypolipidemic agent used as a cholesterol absorption inhibitor. Following an initial fluctuation due to the equilibration for 35 ns, the protein structures RMSD varied between 0.4 Å to 2.7 Å till the end of the simulation (**Fig 2S.E**). The protein structure fluctuations between 2.3 Å indicated a stable protein structure where the complex has not undergone significant conformational changes. Similarly, the ligand structures RMSD varied between 1.9 Å to 3.2 Å till the end of the simulation. The ligand structure fluctuations remained between 1.3 Å, indicating that the ligand is stably bound to the kinase allosteric site and has not diffused significantly from the bound position. **Fig 7S** depicts the ligand-protein interactions as H-bonds, hydrophobic, ionic, and water bridges were observed as protein-ligand contacts recorded throughout the MD simulation over 30% of the simulation time. The 4-hydroxyphenyl side group in Ezetimibe demonstrated a π-π interaction with Trp-80, which persisted for 78% of the simulations. The hydroxyl group in the same ring demonstrated a water-bridged hydrogen bonding interaction with the Asp-292, which persisted for 47% of the simulations. The hydroxyl functional group in the 3-hydroxypropyl chain demonstrated a direct hydrogen bonding interaction with the Tyr-272 residue that persisted for 32% of the simulations. The amino acid residues, Asn-54 and Leu-264, demonstrated hydrophobic interactions with the ligand. The residue Trp-80 demonstrated frequent contact with the ligand throughout the simulation. The RMSD plot of Ezetimibe shows peaks of around ~1.638 Å. The radius of gyration measured for the extendedness of the ligand, equivalent to its moment of inertia, shows peaks of around 5.272 Å. The MolSA was around 367.668 to 383.937 Å. The SASA and PSA ranged from 20.66 to 113.101 and 101.254 to 141.262 Å, respectively.

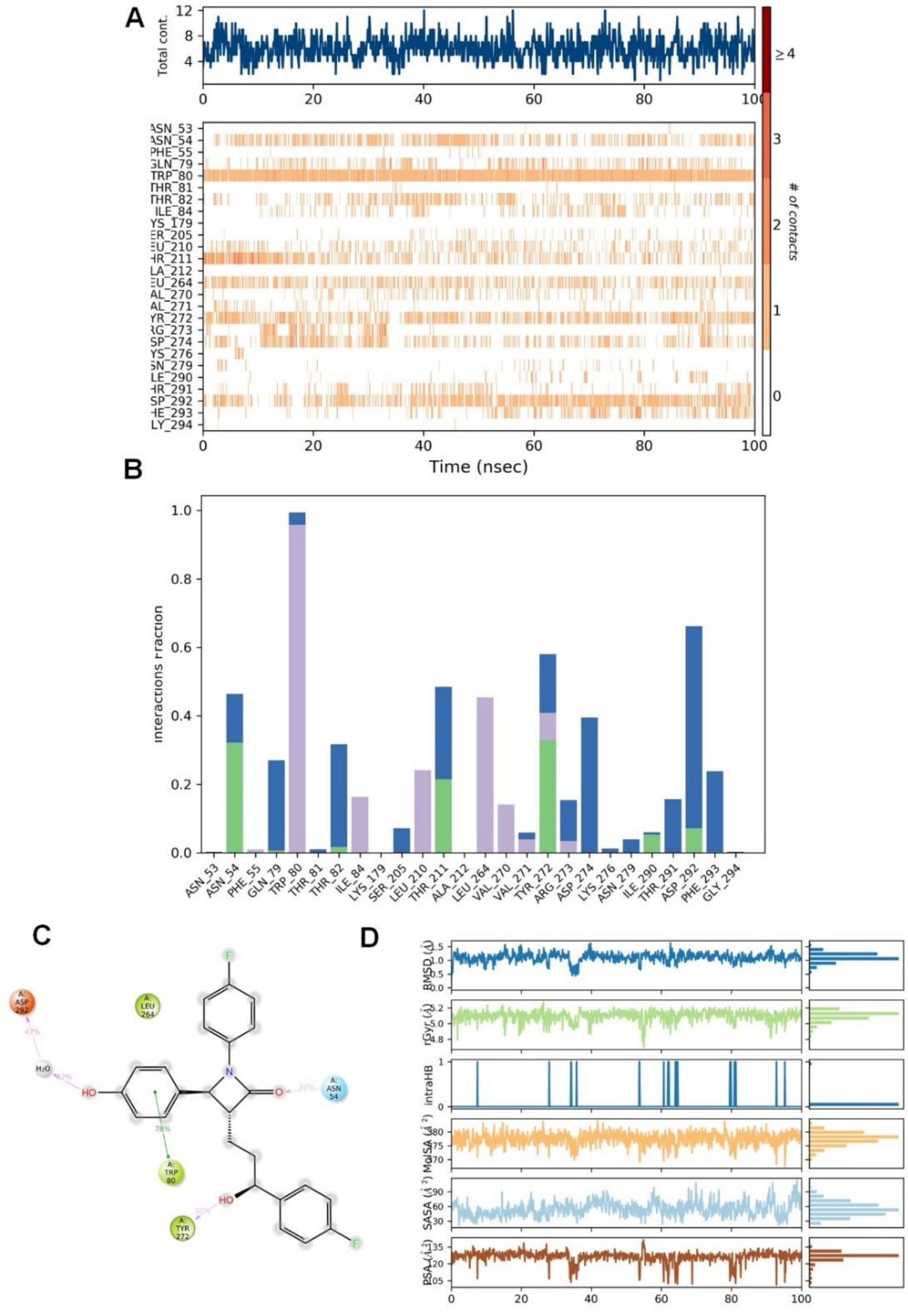

Fig 7S. Protein interactions with the ezetimibe monitored during the course of trajectory (A) Timeline illustration of total specific contacts that several interacting residues makes with a ligand (B) Protein- ligand contacts described as histogram (C) Various protein- ligand interactions that stays for more than 30.0% of the simulation time (D) Various ligand properties in the form of ligand RMSD, radius of gyration (rGyr), molecular surface area (MolSA), solvent-accessible surface area (SASA) and polar surface (PSA) expressed in Å during the 100 ns of the MD simulation

Ditazole is an analgesic and antipyretic drug. Following an initial fluctuation due to the equilibration for 35 ns, the protein structures RMSD varied between 0.9 Å to 3.2 Å till the end of the simulation (**Fig 2S.F**). The protein structure fluctuations between 2.3 Å indicated a relatively unstable protein structure where the complex has undergone significant conformational changes. Similarly, the ligand structures RMSD varied between 1.2 Å to 2.5 Å till the end of the simulation. The ligand structure fluctuations remained between 1.3 Å, indicating that the ligand is bound to the kinase allosteric site and has diffused significantly from the bound position. **Fig 8S** depicts the ligand-protein interactions as H-bonds, hydrophobic, ionic, and water bridges were observed as protein-ligand contacts recorded throughout the MD simulation over 30% of the simulation time. The oxazole nitrogen demonstrated a direct hydrogen bonding interaction with the Gln-79 residue, which persisted for 67% of the simulations. The phenyl ring attached to position-5 of the oxazole ring in Ditazole demonstrated a π-π interaction with Trp-80, which persisted for 41% of the simulations. The terminal hydroxyl group in the 2-hydroxyethyl chain of Ditazole showed a direct hydrogen bonding interaction with the Asp-292 residue, which persisted for 88% of the simulation. The idues Thr-82 and Asp-292 demonstrated frequent contact with the ligand. The RMSD plot of Nomegestrol shows peaks of around ~1.097 Å. The radius of gyration measured for the extendedness of the ligand, equivalent to its moment of inertia, shows peaks of around 3.915 Å. The MolSA was around 306.068 to 322.797 Å. The SASA and PSA ranged from 8.323 to 86.46 and 65.428 114.922, respectively.

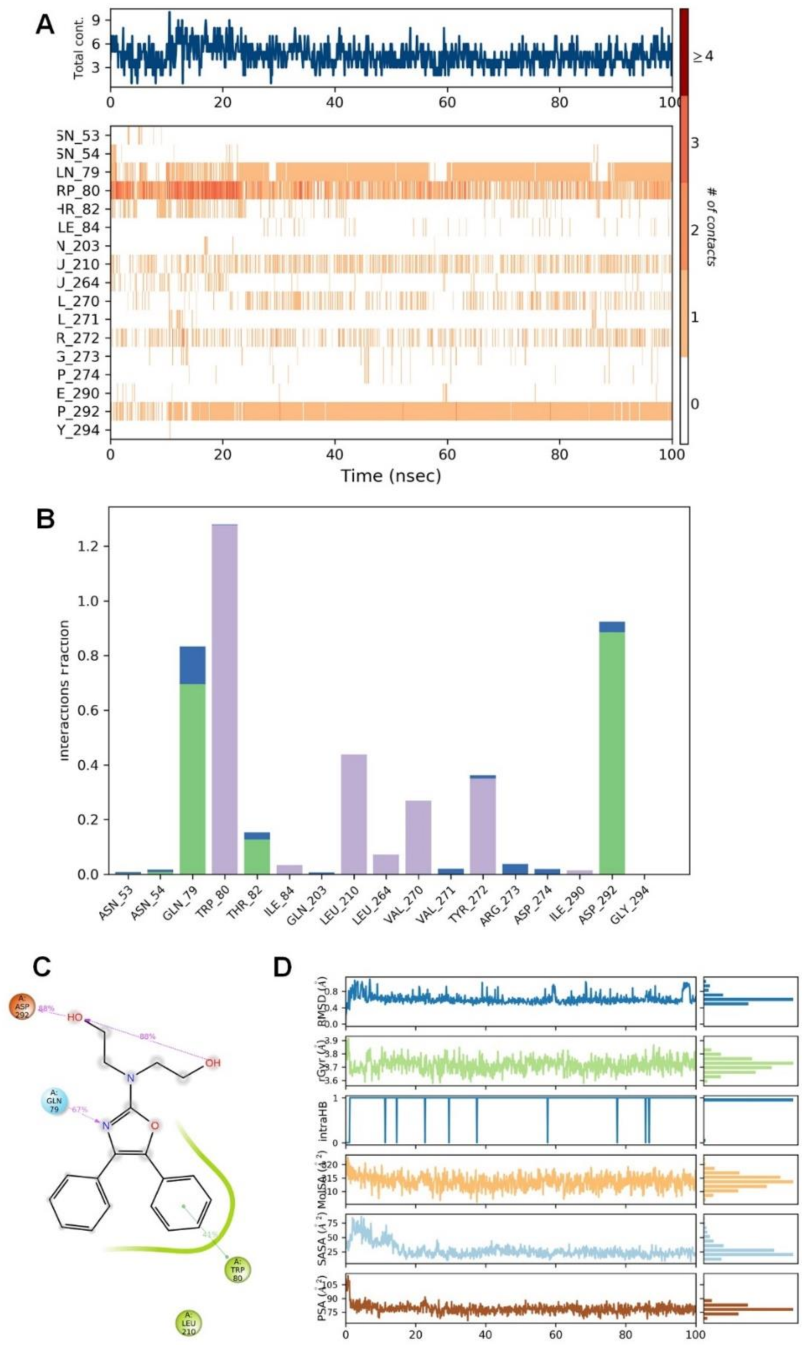

Fig 8S. Protein interactions with the ditazole monitored during the course of trajectory (A) Timeline illustration of total specific contacts that several interacting residues makes with a ligand (B) Protein- ligand contacts described as histogram (C) Various protein- ligand interactions that stays for more than 30.0% of the simulation time (D) Various ligand properties in the form of ligand RMSD, radius of gyration (rGyr), molecular surface area (MolSA), solvent-accessible surface area (SASA) and polar surface (PSA) expressed in Å during the 100 ns of the MD simulation

Nebivolol is an anti-hypertensive drug used in the management of heart failure. Following an initial fluctuation due to the equilibration for 35 ns, the protein structures RMSD varied between 1.0 Å to 2.9 Å till the end of the simulation (**Fig 2S.G**). The protein structure fluctuations between 1.9 Å indicated a stable protein structure where the complex has not undergone significant conformational changes. Similarly, the ligand structures RMSD varied between 1.3 Å to 1.5 Å till the end of the simulation. The ligand structure fluctuations remained between 0.3 Å, indicating that the ligand is stably bound to the kinase allosteric site and has not diffused significantly from the bound position. **Fig 9S** depicts the ligand-protein interactions as H-bonds, hydrophobic, ionic, and water bridges were observed as protein-ligand contacts recorded throughout the MD simulation over 30% of the simulation time. One chromene ring in Nebivolol that contains the 6-fluoro functional group demonstrated a π-π interaction with Trp-80, which persisted for 86% of the simulation. The hydroxyl functional groups in the 2-hydroxyethyl groups of Nebivolol demonstrate direct hydrogen bonding interactions with residues Thr-82, Asp-292 and Asp-274, which persisted for 30, 76 and 91% of the simulations. The amino functional group at the junction of two 2-hydroxyethyl groups demonstrated direct hydrogen bonding interactions with residues Asp-274 and Asp-292, which persisted for 87 and 78% of the simulations. Asp-274, Asp-292 and Trp-80ues demonstrated frequent contact with the ligand. The RMSD plot of nebivolol shows peaks of around ~1.031 Å. The radius of gyration measured for the extendedness of the ligand, equivalent to its moment of inertia, shows peaks of around 5.952 Å. The MolSA was around 370.068 to 383.707 Å. The SASA and PSA ranged from 4.588 to 58.308 and 91.131 to 114.351 Å, respectively.

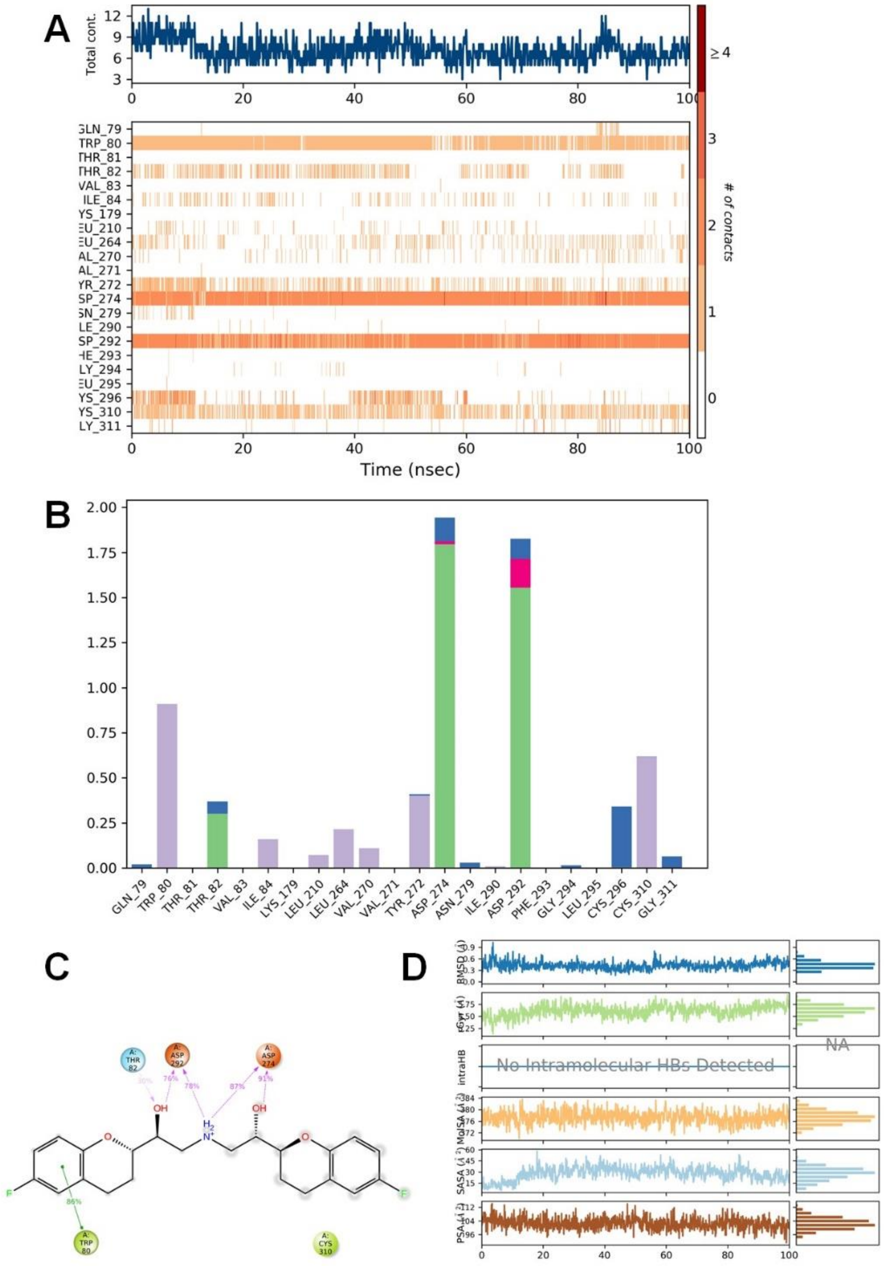

Fig 9S. Protein interactions with the nebivolol monitored during the course of trajectory (A) Timeline illustration of total specific contacts that several interacting residues makes with a ligand (B) Protein- ligand contacts described as histogram (C) Various protein- ligand interactions that stays for more than 30.0% of the simulation time (D) Various ligand properties in the form of ligand RMSD, radius of gyration (rGyr), molecular surface area (MolSA), solvent-accessible surface area (SASA) and polar surface (PSA) expressed in Å during the 100 ns of the MD simulation

Floxuridine is an anti-cancer drug used in the management for liver metastases from gastrointestinal cancers. After the initial fluctuation due to the equilibration for 35 ns, the protein structures RMSD varied between 0.9 Å to 3.2 Å till the end of the simulation (**Fig**

2S.H). The protein structure fluctuations between 2.3 Å indicated a stable protein structure where the complex has not undergone significant conformational changes. Similarly, the ligand structures RMSD varied between 1.6 Å to 2.8 Å till the end of the simulation. The ligand structure fluctuations remained between 1.2 Å, indicating that the ligand is stably bound to the kinase allosteric site and has not diffused significantly from the initially bound position. **Fig 10S** depicts the ligand-protein interactions observed as protein-ligand contacts recorded throughout the MD simulation over 30% of the simulation time. The 4-hydroxy functional group attached to the tetrahydrofuran/oxolan ring demonstrated direct hydrogen bonding interactions with Tyr-18 and Asp-274 residues that persisted for 98% of the simulation. The 2-oxo functional group in the pyrimidine-2,4-dione showed intramolecular hydrogen bonding interactions with the 5-hydroxyethyl functional group, which persisted for 61% of the simulations. The 4-oxo functional group in the pyrimidine-2,4-dione ring of floxuridine demonstrated direct hydrogen bonding interactions with residues, Glu-85 and Glu-298, that persisted for 97 and 56% of simulations. The N-3 of the pyrimidine-2,4-dione displayed a direct hydrogen bonding interaction with Cys-296 residue that persisted for 88% of the simulations. The Cys-296 residue demonstrated water-bridged hydrogen bonding interactions with the 4-oxo functional group of the pyrimidine-2,4-dione ring. The residues Tyr-18, Asp-274, Glu-85 and Cys-296 made frequent contact with the ligand throughout the simulation. The RMSD plot of floxuridine shows peaks of around ~1.276 Å. The radius of gyration measured for the extendedness of the ligand, equivalent to its moment of inertia, shows peaks of around 3.22 Å. The MolSA was around 202.41 to 215.154 Å. The SASA and PSA ranged from 7.979 to 75.091 and 176.979 to 217.931 Å, respectively.

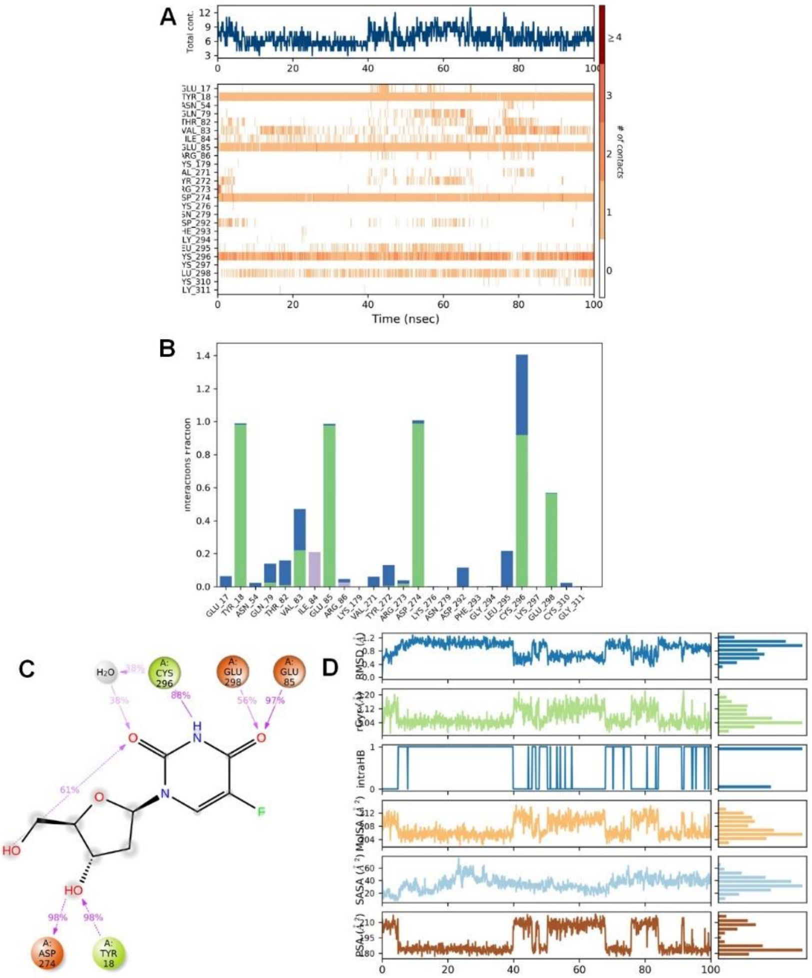

Fig 10S. Protein interactions with the floxuridine monitored during the course of trajectory (A) Timeline illustration of total specific contacts that several interacting residues makes with a ligand (B) Protein- ligand contacts described as histogram (C) Various protein- ligand interactions that stays for more than 30.0% of the simulation time (D) Various ligand properties in the form of ligand RMSD, radius of gyration (rGyr), molecular surface area (MolSA), solvent-accessible surface area (SASA) and polar surface (PSA) expressed in Å during the 100 ns of the MD simulation

Delorazepam is an anxiolytic and anti-insomnia drug. Following an initial fluctuation due to the equilibration for 35 ns, the protein structures RMSD varied between 1.2 Å to 2.8 Å till the end of the simulation (**Fig 2S.I**). The protein structure fluctuations between 2.3 Å indicated a stable protein structure where the complex has not undergone significant conformational changes. Similarly, the ligand structures RMSD varied between 1.2 Å to 1.7 Å till the end of the simulation. The ligand structure fluctuations remained between 0.5 Å, indicating that the ligand is stably bound to the kinase allosteric site and has not diffused significantly from the

bound position. **Fig 11S** depicts the ligand-protein interactions as H-bonds, hydrophobic, ionic, and water bridges were observed as protein-ligand contacts recorded throughout the MD simulation over 30% of the simulation time. The 2-chlorophenyl ring in Delorazepam demonstrated a π-π interaction with the Trp-80 residue, which persisted for 69% of the simulations. The benzodiazepine ring demonstrated direct hydrogen bonding interactions during the simulations. The N-4 and 2-oxo functional groups in the benzodiazepine ring demonstrated direct hydrogen bonding interactions with Thr-211 residue, accounting for 80 and 96% of the simulations. The residues Thr-211 and Trp-80 demonstrated frequent contact with the ligand throughout the simulation. The RMSD plot of Delorazepam shows peaks of around ~0.415 Å. The radius of gyration measured for the extendedness of the ligand, equivalent to its moment of inertia, shows peaks of around 3.325 Å. The MolSA was around 252.818 to 261.121 Å. The SASA and PSA ranged from 6.216 to 76.459 and 72.005 to 82.471 Å, respectively.

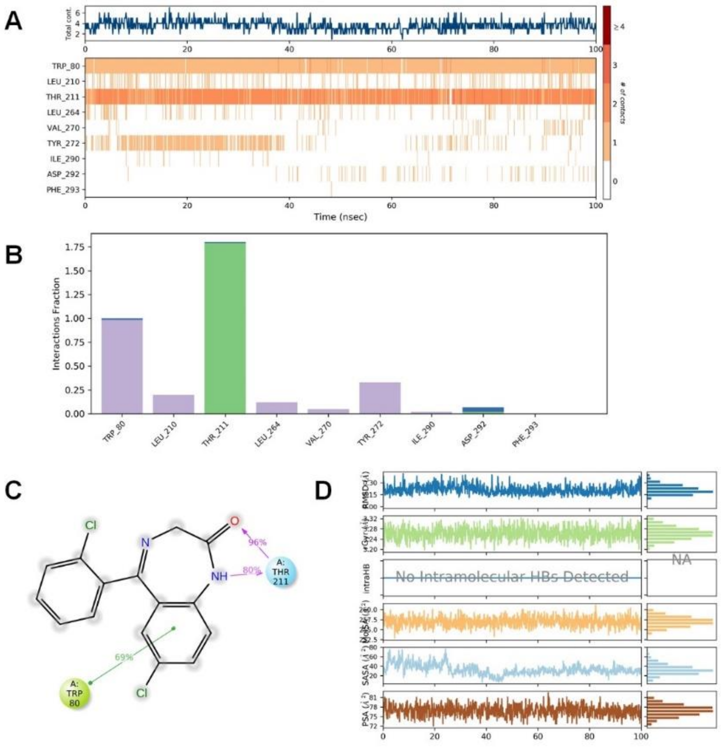

Fig 11S. Protein interactions with the delorazepam monitored during the course of trajectory (A) Timeline illustration of total specific contacts that several interacting residues makes with a ligand (B) Protein- ligand contacts described as histogram (C) Various protein- ligand interactions that stays for more than 30.0% of the simulation time (D) Various ligand properties in the form of ligand RMSD, radius of gyration (rGyr), molecular surface area (MolSA), solvent-accessible surface area (SASA) and polar surface (PSA) expressed in Å during the 100 ns of the MD simulation

Lorazepam is an anxiolytic and anti-seizure drug. Following an initial fluctuation due to the equilibration for 35 ns, the protein structures RMSD varied between 1.0 Å to 3.5 Å till the end of the simulation (**Fig 2S.J**). The protein structure fluctuations between 2.5 Å indicated a stable protein structure where the complex has not undergone significant conformational changes. Similarly, the ligand structures RMSD varied between 2.1 Å to 3.2 Å till the end of the simulation. The ligand structure fluctuations remained between 1.1 Å, indicating that the ligand is stably bound to the kinase allosteric site and has not diffused significantly from the bound position. **Fig 12S** depicts the ligand-protein interactions as H-bonds, hydrophobic, ionic, and water bridges were observed as protein-ligand contacts recorded throughout the MD simulation over 30% of the simulation time. The N-4 of the benzodiazepine ring of lorazepam demonstrated water-bridged hydrogen bonding interactions with Asp-274, which persisted for 76% of simulations. The 2-oxo functional group of benzodiazepine demonstrated direct hydrogen bonding interaction with Asp-274 residue that persisted for 62 and 36% of the simulations. The 2-oxo functional group in the benzodiazepine ring in lorazepam demonstrated direct hydrogen bonding interactions with Asn-274, Phe-293 and Gly-294 that persisted for 49, 76 and 47% of the interactions. The N-4 of the benzodiazepine ring demonstrated a direct hydrogen bonding interaction with Asp-292 residue that persisted for 63% of the simulations. The residues Trp-80, Tyr-272 and Cys-310, demonstrated hydrophobic interactions with the ligand. The residues Asp-274 and Asn-279 made frequent contact with the ligand. The RMSD plot of lorazepam shows peaks of around ~0.53 Å. The radius of gyration measured for the extendedness of the ligand, equivalent to its moment of inertia, shows peaks of around 3.401 Å. The MolSA was around 259.997 to 268.257 Å. The SASA and PSA ranged from 1.546 to 53.39 and 108.815 to 127.593 Å, respectively.

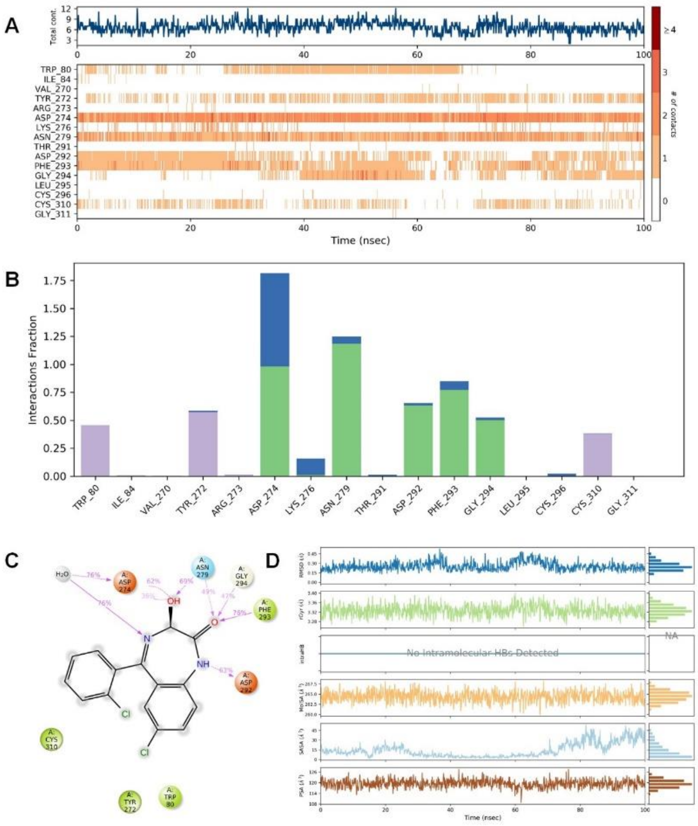

Fig 12S. Protein interactions with the lorazepam monitored during the course of trajectory (A) Timeline illustration of total specific contacts that several interacting residues makes with a ligand (B) Protein- ligand contacts described as histogram (C) Various protein- ligand interactions that stays for more than 30.0% of the simulation time (D) Various ligand properties in the form of ligand RMSD, radius of gyration (rGyr), molecular surface area (MolSA), solvent-accessible surface area (SASA) and polar surface (PSA) expressed in Å during the 100 ns of the MD simulation
