# Supplementary material for: Computational drug repurposing of Akt-1 allosteric inhibitors for non-small cell lung cancer
Source: Sci Rep. 2023 May 16;13:7947. doi: 10.1038/s41598-023-35122-7 (PMC10188557; doi:10.1038/s41598-023-35122-7)
Supplement: Supplementary file 2 — Supplementary Information 2. [file 41598_2023_35122_MOESM2_ESM.pptx]

## Slide 1
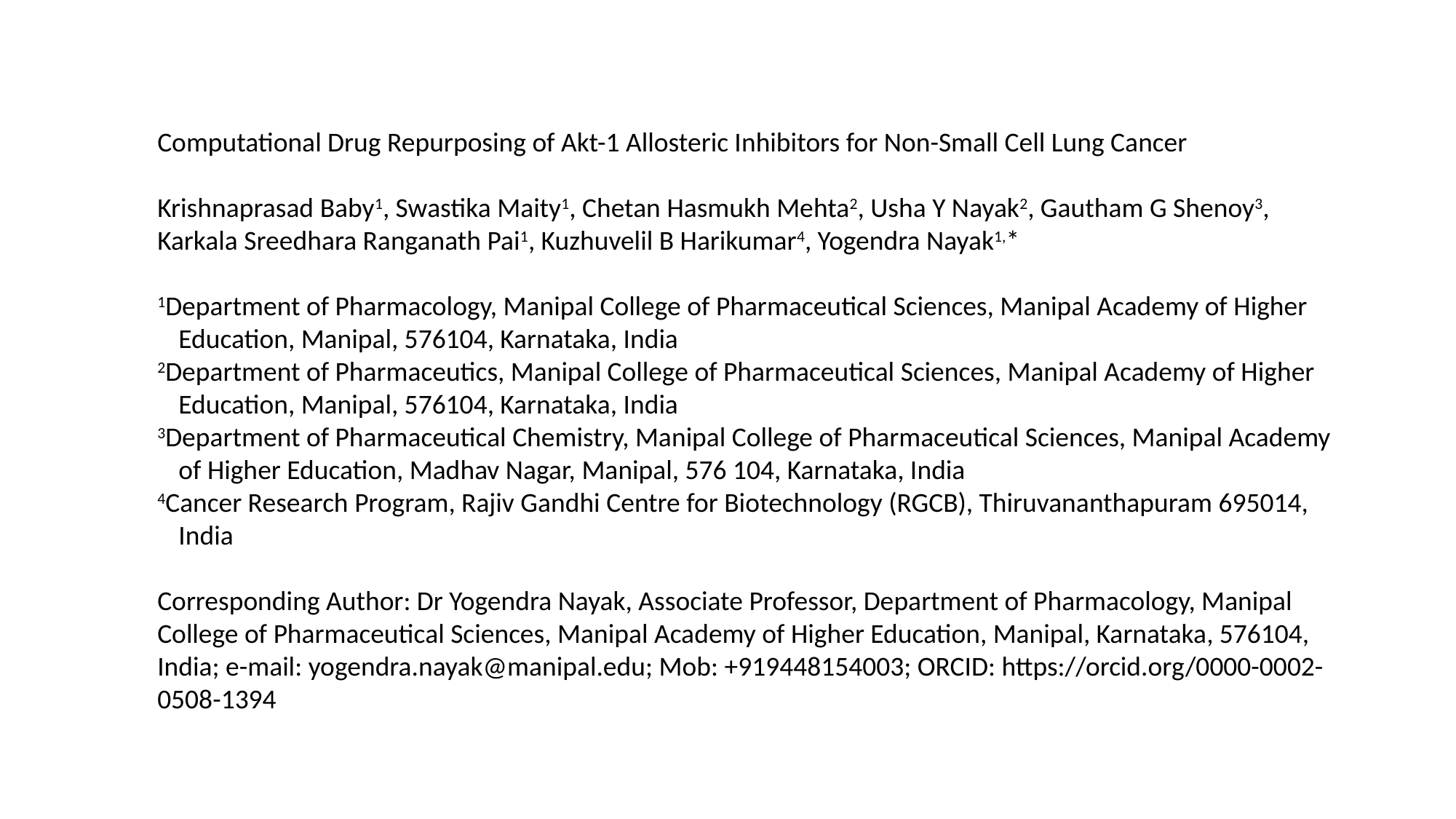

Computational Drug Repurposing of Akt-1 Allosteric Inhibitors for Non-Small Cell Lung Cancer
Krishnaprasad Baby1, Swastika Maity1, Chetan Hasmukh Mehta2, Usha Y Nayak2, Gautham G Shenoy3, Karkala Sreedhara Ranganath Pai1, Kuzhuvelil B Harikumar4, Yogendra Nayak1,*
1Department of Pharmacology, Manipal College of Pharmaceutical Sciences, Manipal Academy of Higher Education, Manipal, 576104, Karnataka, India
2Department of Pharmaceutics, Manipal College of Pharmaceutical Sciences, Manipal Academy of Higher Education, Manipal, 576104, Karnataka, India
3Department of Pharmaceutical Chemistry, Manipal College of Pharmaceutical Sciences, Manipal Academy of Higher Education, Madhav Nagar, Manipal, 576 104, Karnataka, India
4Cancer Research Program, Rajiv Gandhi Centre for Biotechnology (RGCB), Thiruvananthapuram 695014, India
Corresponding Author: Dr Yogendra Nayak, Associate Professor, Department of Pharmacology, Manipal College of Pharmaceutical Sciences, Manipal Academy of Higher Education, Manipal, Karnataka, 576104, India; e-mail: yogendra.nayak@manipal.edu; Mob: +919448154003; ORCID: https://orcid.org/0000-0002-0508-1394

## Slide 2
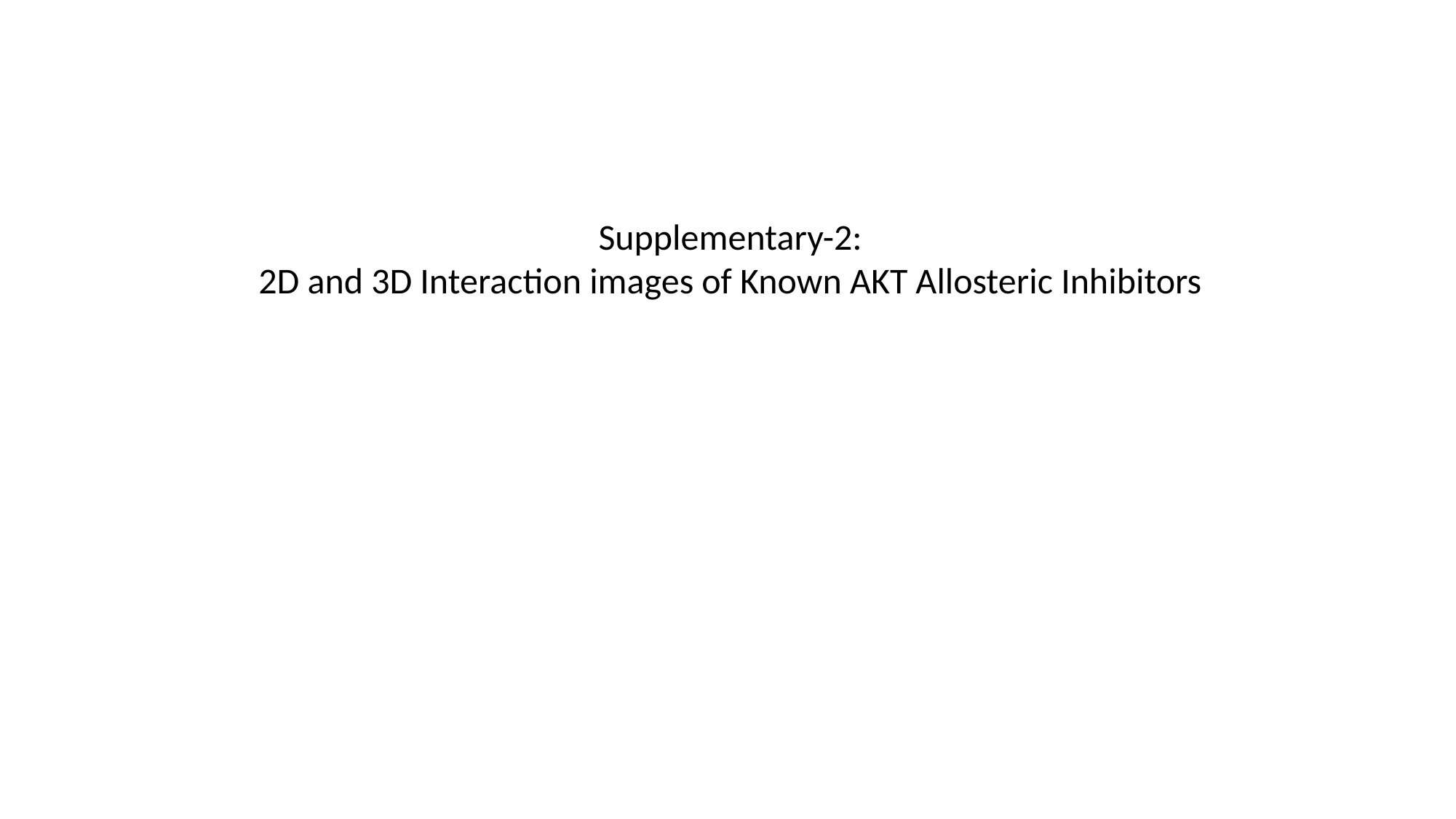

Supplementary-2:
2D and 3D Interaction images of Known AKT Allosteric Inhibitors

## Slide 3
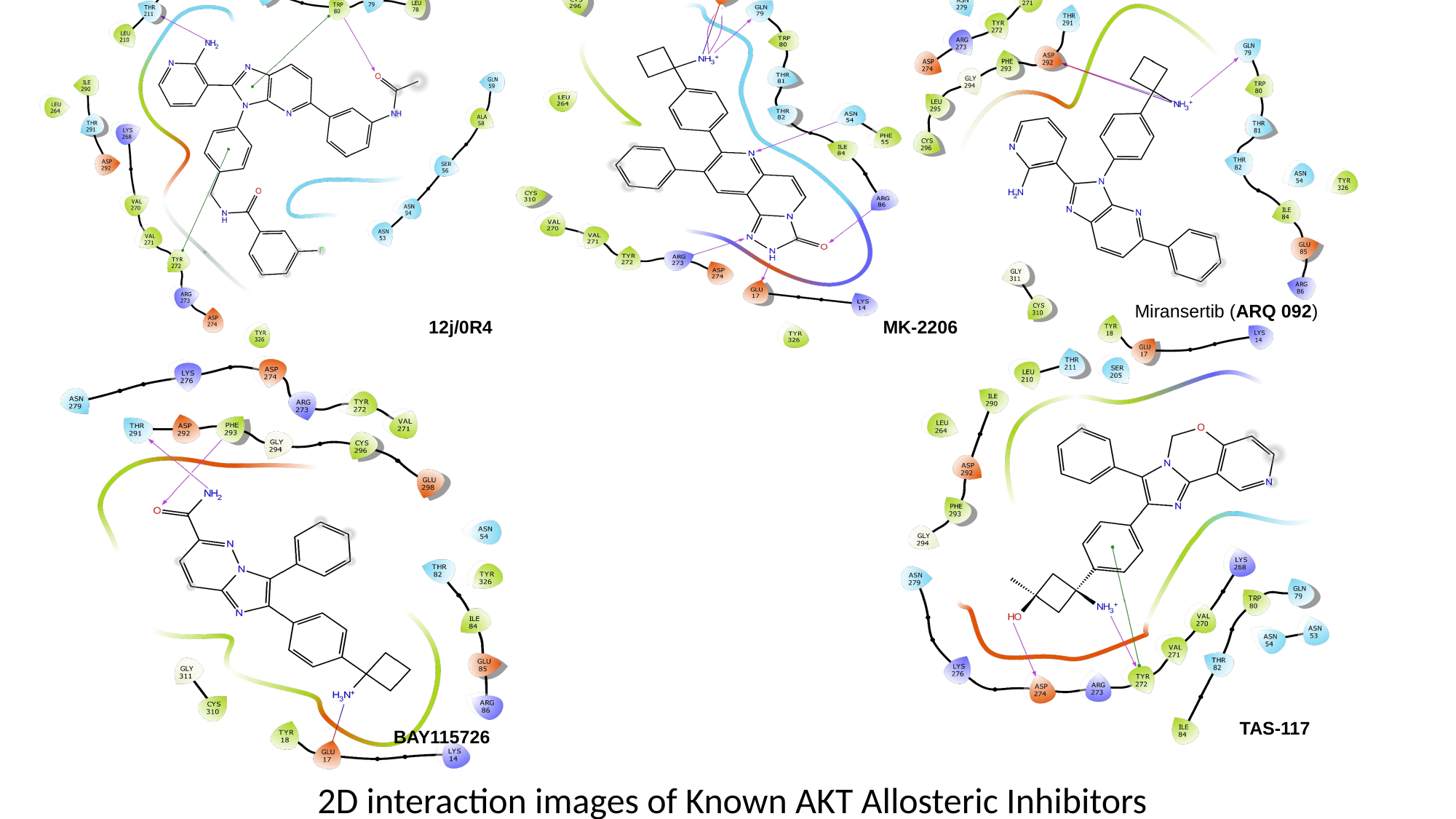

Miransertib (ARQ 092)
MK-2206
12j/0R4
TAS-117
BAY115726
2D interaction images of Known AKT Allosteric Inhibitors

## Slide 4
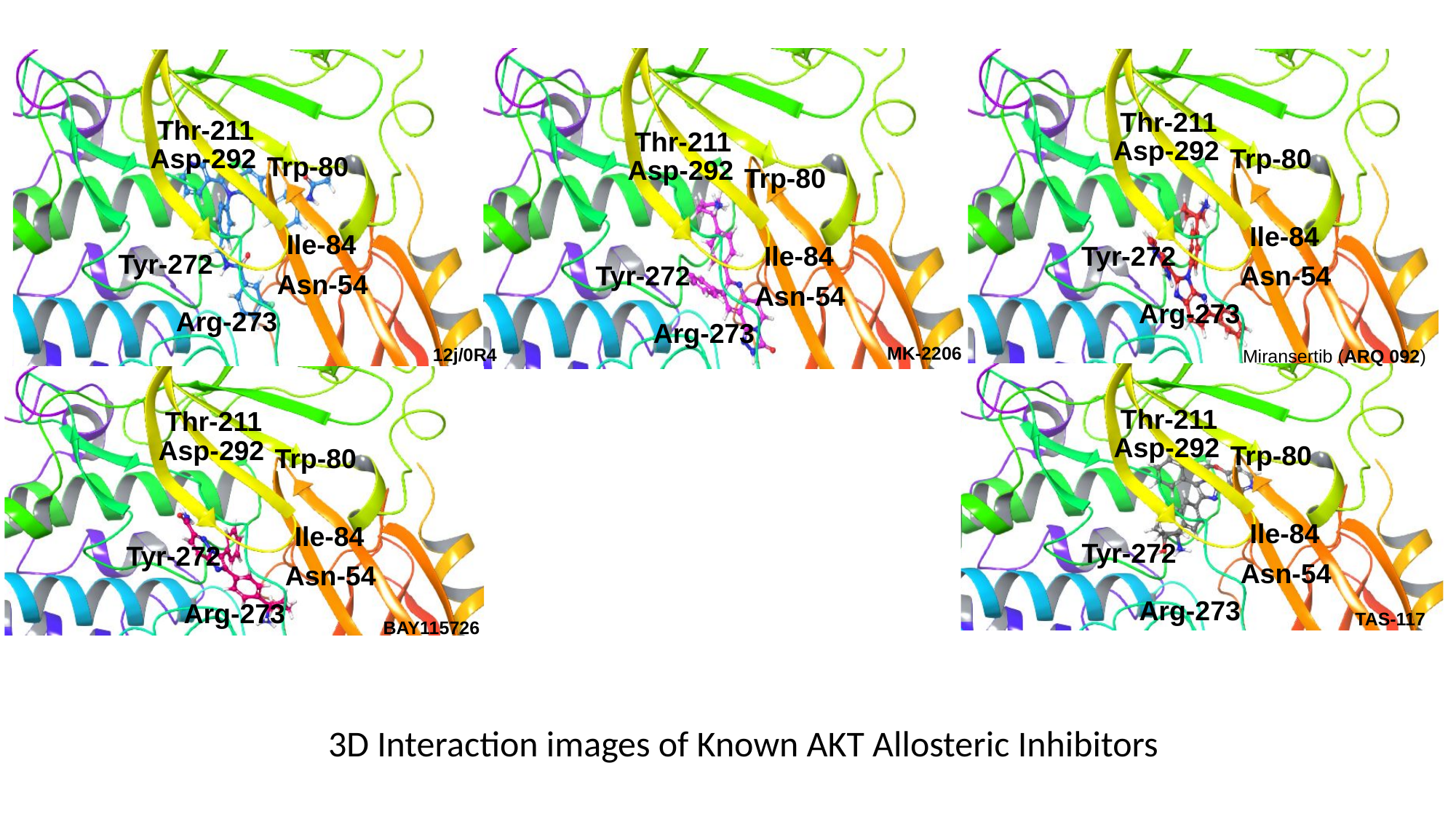

Thr-211
Thr-211
Thr-211
Asp-292
Asp-292
Trp-80
Trp-80
Asp-292
Trp-80
Ile-84
Ile-84
Ile-84
Tyr-272
Tyr-272
Tyr-272
Asn-54
Asn-54
Asn-54
Arg-273
Arg-273
Arg-273
Thr-211
Thr-211
Asp-292
Asp-292
Trp-80
Trp-80
Ile-84
Ile-84
Tyr-272
Tyr-272
Asn-54
Asn-54
Arg-273
Arg-273
TAS-117
BAY115726
MK-2206
12j/0R4
Miransertib (ARQ 092)
3D Interaction images of Known AKT Allosteric Inhibitors
